# Endothelial‐specific telomerase inactivation causes telomere‐independent cell senescence and multi‐organ dysfunction characteristic of aging

**DOI:** 10.1111/acel.14138

**Published:** 2024-03-12

**Authors:** Zhanguo Gao, Rafael Bravo Santos, Joseph Rupert, Rachel Van Drunen, Yongmei Yu, Kristin Eckel‐Mahan, Mikhail G. Kolonin

**Affiliations:** ^1^ The Brown Foundation Institute of Molecular Medicine University of Texas Health Science Center Houston Texas USA

**Keywords:** accelerated aging, endothelial, hypoxia, knockout, metabolism, mitochondrial disease, senescence, telomerase

## Abstract

It has remained unclear how aging of endothelial cells (EC) contributes to pathophysiology of individual organs. Cell senescence results in part from inactivation of telomerase (TERT). Here, we analyzed mice with *Tert* knockout specifically in EC. *Tert* loss in EC induced transcriptional changes indicative of senescence and tissue hypoxia in EC and in other cells. We demonstrate that EC‐*Tert*‐KO mice have leaky blood vessels. The blood–brain barrier of EC‐*Tert*‐KO mice is compromised, and their cognitive function is impaired. EC‐*Tert*‐KO mice display reduced muscle endurance and decreased expression of enzymes responsible for oxidative metabolism. Our data indicate that *Tert*‐KO EC have reduced mitochondrial content and function, which results in increased dependence on glycolysis. Consistent with this, EC‐*Tert*‐KO mice have metabolism changes indicative of increased glucose utilization. In EC‐*Tert*‐KO mice, expedited telomere attrition is observed for EC of adipose tissue (AT), while brain and skeletal muscle EC have normal telomere length but still display features of senescence. Our data indicate that the loss of *Tert* causes EC senescence in part through a telomere length‐independent mechanism undermining mitochondrial function. We conclude that EC‐*Tert*‐KO mice is a model of expedited vascular senescence recapitulating the hallmarks aging, which can be useful for developing revitalization therapies.

## Graphical abstract

This study demonstrates that telomerase in endothelial cells is important for mitochondrial function and vascular integrity. We characterize mice lacking telomerase in endothelial cells, which have senescence and hypoxia induced in perivascular cells. The resulting metabolic dysfunction accelerates aging‐associated pathogenesis in adipose tissue, brain, and the skeletal muscle.

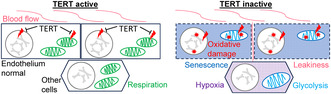

**Abbreviations**
AD: Alzheimer's disease
ASC: adipose stromal cells
AT: adipose tissue
BAT: brown adipose tissue
BBB: blood–brain barrier
CLEAR: coordinated lysosomal expression and regulation
CVD: cardio‐vascular disease
DN: dominant negative
EC: endothelial cells
EdU: 5‐ethynyl‐2′‐deoxyuridine
FFAs: free fatty acids
FISH: fluorescent in situ hybridization
GM: gastrocnemius
HFD: high‐fat diet
HIF1a: hypoxia‐inducible factor‐1 alpha
HRP: horseradish peroxidase
IB4: isolectin B4
IF: immunofluorescence
IP: intraperitoneal
IPA: ingenuity pathway analysis
KO: knockout
LPS: lipopolysaccharide
mG: membrane GFP
mT: membrane Tomato
NORT: novel object recognition test
OCR: oxygen consumption rate
OFT: open field test
qRT‐PCR: quantitative real‐time PCR
Quad: quadriceps
RER: respiratory exchange ratio
RNAseq: RNA sequencing
ROS: reactive oxygen species
SA‐β‐gal: Senescence‐associated β‐galactosidase
SASP: senescence‐associated secretory phenotype
SAT: subcutaneous AT
SDH1: succinate dehydrogenase
scRNAseq: single cell RNA sequencing
SMC: smooth muscle cells
SVC: stromal/vascular cells
TERT: telomerase
TRF2: telomeric repeat‐binding factor 2
VAT: visceral AT
WAT: white adipose tissue

## 1 INTRODUCTION

Through lifetime, chromosomal and mitochondrial DNA, as well as other cellular macromolecules, accumulate damage, which, when unresolved, culminates in cell senescence (Baker et al., 2023), a hallmark of aging (Lopez‐Otin et al., 2022). Cell senescence is characterized by cell cycle arrest, which is often accompanied by pro‐inflammatory senescence‐associated secretory phenotype (SASP), as well as by expression of senescence markers including p53 (coded by *Tp53*), p16 (coded by *Cdkn2a*), p21 (coded by *Cdkn1a*), and β‐galactosidase (Liu et al., 2019; Zhang et al., 2022). Accumulation of senescent cells is responsible for tissue changes leading to aging‐related diseases (Borghesan et al., 2020; Gorgoulis et al., 2019). Senescent cells have a bystander effect on surrounding cells that often acquire a secondary senescence‐like phenotype (Yousefzadeh et al., 2021). Senolytic and senomorphic interventions have been shown to delay the manifestations of aging in mouse models (Baker et al., 2011; Palmer et al., 2019).

Double‐stranded DNA breaks due to genotoxic stress anywhere in the genome can cause cell senescence (Baker et al., 2023; Borghesan et al., 2020). However, replicative senescence particularly often results from damage to telomeres and from telomere shortening to a critical limit of ~5 kb (Chakravarti, LaBella, & DePinho, 2021; Whittemore et al., 2019). Telomerase Reverse Transcriptase (TERT) protein is the catalytic subunit of the enzyme complex that lengthens telomeres to prevent their attrition (Blackburn et al., 2006; Blasco, 2007). TERT is also implicated in protection from genotoxic stress (Sahin & Depinho, 2010) and has genome‐wide telomere‐independent effects on cell transcriptome and physiology (Mojiri et al., 2021). Recent reports have shown that TERT localizes to mitochondria, where it functions to mitigate oxidate stress (Ait‐Aissa et al., 2022; Ale‐Agha et al., 2021). In humans, TERT is active in stem cells, but is turned off in early development, which opens the path to telomere erosion and cell aging. There is accumulating evidence that inactivation of TERT and of the multi‐pronged cytoprotective mechanisms regulated by it is the key contributor to cell aging leading to senescence (Chakravarti, LaBella, & DePinho, 2021).

To develop safe and effective approaches targeting senescence, consideration needs to be given to specific cell types and better understanding of the roles they play in disease pathogenesis. Accumulation of senescent cells is particularly important to consider for the vasculature, the lining of cells exposed to high glucose and lipids, which are elevated in metabolic disease and impose a stress on the endothelium (Donato et al., 2018). Vascular endothelial cells (EC) are also the first line of defense against xenobiotics. Endothelial senescence has been implicated in various age‐related cardiovascular diseases (CVDs) including atherosclerosis, stroke, myocardial infarction, peripheral artery disease, and type‐2 diabetes (Dominic et al., 2020; Han & Kim, 2023). The resulting inflammation and oxidative stress, increasing in the endothelium during aging, are the likely culprits of vascular deterioration (Bloom et al., 2022). For example, neurodegenerative diseases, including Alzheimer's disease (AD) and other dementias, are linked with accumulation of senescent cells in the brain (Ogrodnik et al., 2021). Vascular dementia, due to abnormal blood flow, is the second most common form of dementia (Romay et al., 2019). Endothelial and perivascular cells are pivotal in the maintenance of the blood–brain barrier (BBB), and their dysfunction in aging sets the stage for inflammatory processes known to contribute to vascular dementia (Sweeney et al., 2018). Accumulation of senescent cells in adipose tissue (AT) is accelerated in obesity (Ogrodnik et al., 2019), the condition predisposing to aging‐related CVDs. However, it remains unclear which cells in organs become senescent first. There is evidence that EC senescence affects other components of the vascular system, the perivascular/smooth muscle cells, and the parenchyma (Fossel et al., 2022; Han & Kim, 2023). The resulting concerted cell dysfunction leads to inefficient vasodilation and obstructed blood flow (Donato et al., 2018), which sets the stage for cardiovascular and degenerative disease (Xu et al., 2022). We have reported that accelerated telomere shortening in perivascular progenitor cells in mice predisposes to AT dysfunction (Gao et al., 2020). However, the potential priming role of EC senescence in AT and other organs, and its effect on the development of metabolic and other diseases, has not been investigated.

In distinct organs, EC are exposed to different microenvironmental pressures and, hence, have different rates of proliferation and self‐renewal leading to telomere shortening and replicative senescence (Fossel et al., 2022). The lack of progress in our understanding of EC senescence mechanisms and repercussions in distinct organs is in part due to laboratory mice being suboptimal as a model of replicative senescence. While humans are born with telomeres in a 10–15 kb range, mice of the C57BL/6 background typically used are born with telomeres of 50–100 kb and continue to express TERT in somatic cells (Kipling & Cooke, 1990; Prowse & Greider, 1995). Their resistance to replicative senescence is illustrated by telomerase inactivation being necessary for the manifestation of Duchenne muscular dystrophy symptoms in dystrophin mutant mice (Sacco et al., 2010). As we have reported, knockout (KO) of *Tert* gene in progenitor cells of *Pdgfra + or Pdgfrb +* lineage leads to cell senescence in AT of mice fed high‐fat diet, which induces excessive proliferation and exhaustion of progenitor cells (Ribas‐Latre et al., 2021) and results in type‐2 diabetes (Gao et al., 2020).

Here, to test the requirement of TERT for endothelial function, we generated mice with *Tert* KO specifically in EC. We show that in EC‐*Tert*‐KO mice developing diet‐induced obesity, telomere attrition is accelerated in EC of AT. Although telomere length remained normal in the brain and skeletal muscle of aging EC‐*Tert*‐KO mice, EC still displayed features of senescence. We have characterized organ‐specific effects of *Tert*‐KO on EC proliferation, differentiation, and function of the vasculature. We report metabolic, cognitive, and skeletal muscle impairment phenotypes indicative of the TERT role in EC mitochondrial function. Our data indicate that the EC‐*Tert*‐KO model is representative of many aspects of aging‐associated organ dysfunction.

## 2 MATERIALS AND METHODS

### 2.1 Mouse experiments

All animal studies were in compliance with the Guide and Use of Laboratory Animals and were approved by the UTHealth Animal Care and Use Committee. Mice were housed in the animal facility with a 12‐h light/dark cycle and constant temperature (22–24°C). *Tert* ^fl/fl^ mice, *Tie2e‐Cre*, and *mT/mG* reporter (Jackson Stock No. 007676) strains have been characterized previously (Kano et al., 2003; Liu et al., 2015; Muzumdar et al., 2007). PCR‐based genotyping was performed as we previously described (Daquinag et al., 2021, 2022; Gao et al., 2020, 2022). In experiments done for both sexes, the same effects were observed in both males and females. For obesity induction, mice were fed 45 kcal% (fat) diet (Research Diets, D12451). Body composition was measured by EchoMRI‐100T (Echo Medical Systems). Glucose and insulin tolerance tests were performed as described in our previous studies (Daquinag et al., 2021, 2022; Gao et al., 2020, 2022). Indirect calorimetry data and food intake were quantified over a 3‐day time course in Phenomaster metabolic chambers (TSE Systems). Core body temperature was determined in the rectum at 2.5 cm in depth using a MicroTherma 2 K High Precision Type K Thermocouple Meter (ThermoWorks, THS‐221‐092) with RET‐3 probe (Braintree Scientific). Cold tolerance/adaptive thermogenesis was measured upon placing mice into environmental chamber IS33SD (Powers Scientific) as described (Gao et al., 2018). Open field test (OFT) for spontaneous locomotor activity was performed for 10 min with undisturbed behavior monitored continuously using the system Kinder Scientific Motor Monitor (Kinder Scientific). Animals were placed in the arena (25 × 25 inches), and activity was detected using multiple crossed perpendicular IR beams. In this system, locomotor activity is detected when a beam is interrupted and then data are logged in a computer for further analyses. To analyze activity in the arena, we considered total time (s) in the center, total time (s) in the periphery, and the center/periphery ratio. The open field arena was sanitized with 70% ethanol before every test. For novel object recognition test (NORT), animals were individually exposed to two objects for which the animal showed no preference for 7 min. After preliminary habituation to old objects, animals were moved to a new cage in which the flow cytometry tube was substituted by a small toy (novel object) while a new falcon top (familiar object) was placed. Mice were recorded for 7 min in this new situation in order to quantify the time they closely explored the novel and the familiar objects. The interaction with the objects were quantified with the free and open‐source event‐logging software BORIS. After data were exported, the discrimination index was calculated. Results were expressed as total time exploring novel and familiar object and discrimination index as the time ratios spent with the novel object / both objects. A grip strength meter with a single sensor for mice along with standard pull bars and software (Columbus Instruments, 1027SM) was used to measure grip strength. For endurance assessment, Exer‐3/6 Treadmill 1055‐SDRMAI‐D60 equipped with shock detection/auto‐calibration was used. Mice were run at 12.5 m/s for exhaustion or at 9.8 m/s to measure fatigue resistance. Lipopolysaccharide (LPS) was administered intraperitoneally (IP) 16 weeks prior to NORT at a dose of 500 μg/kg BW.

### 2.2 Tissue and cell analysis

Evans blue (vascular permeability probe) was injected via tail vein and, after cardiac perfusion with 10 mL PBS, tissue retention was measured a described (Serghides et al., 2011). To measure hypoxia, Hypoxyprobe (60 mg/kg BW) was injected IP as described (Ye et al., 2007). Inguinal AT was analyzed as SAT, and gonadal AT was analyzed as VAT. Angiogenic sprouting assay with mouse AT explants was performed as previously described (Min et al., 2016; Salameh et al., 2016) in EGM‐2MV medium (Lonza, CC‐4147). For cell proliferation measurement, mice were IP‐injected with 100 mg/kg EdU 3 days prior to necropsy to measure the frequency of EdU+ populations by flow cytometry as described (Gao et al., 2020). AT cell suspensions were isolated as described (Daquinag et al., 2011, 2015; Gao et al., 2018): minced AT was digested in 0.5 mg/mL collagenase type I (Worthington Biochemical) and 2.5 mg/mL of dispase (Roche, 04942078001) solution under gentle agitation for 1 h at 37°C and centrifuged at 400 *g* for 5 min to separate the stromal/vascular pellet from the adipocytes. The SVF were plated in 8‐well chamber slides (Thermo Fisher, 154941) or 12‐well plates in DMEM/10% FBS. The Seahorse XF Cell Mito Stress Test Kit (Agilent Technologies, 103015‐100) was used to analyze mitochondrial respiration. The oxygen consumption rate (OCR) was measured upon successive treatment with 1 mmol/L oligomycin, 1 mmol/L FCCP, and 0.5 mmol/L rotenone/antimycin A.

### 2.3 Histology and immunoassays

SA‐β‐gal expression in tissues and cultured cells was measured as described (Gao et al., 2020). Brain and muscle, snap‐frozen mounted blocks were serial‐sectioned (10 μm) at −20°C on a Leica CM1860 cryostat and mounted to Superfrost Plus microscope slides (Thermo Fisher 12–550‐15). Myosin IIa and IIb antibodies (DSHB at U Iowa, SC‐71 and BF‐F3) were previously described and used with Donkey Cy5‐conjugated secondary IgG (1:200, Jackson ImmunoResearch). AT whole mounts were prepared as described (Gao et al., 2018). AT formalin‐fixed paraffin‐embedded tissue sections (5 μm) were analyzed by immunofluorescence (IF) upon antigen retrieval as described (Daquinag et al., 2011, 2015; Gao et al., 2018). Upon blocking, GFP: Gene Tex GTX26673 (1:250) or GLUT1: Mybiosourse MBS179154 (1:100) primary antibodies (4°C, 12 h) were used followed by Donkey Alexa488‐conjugated (Invitrogen A11055, 1:200) or Cy3‐conjugated (Jackson ImmunoResearch 711‐166‐152, 1:200) IgG. Biotinylated isolectin B4 (Vector B‐1205) was used at 1:50 and detected with streptavidin‐ Alexa488 (Life Technologies S32354, 1:200). MitoTracker® Deep Red (Molecular Probes, M22426) was used at 0.02 mM. Nuclei were stained with Hoechst 33258 (Invitrogen, H3569). SDH1 activity was measured as described (Rupert et al., 2021). Briefly, muscle sections were submerged in succinic acid (50 mM) solution for 30 min at 37°C and rinsed in 30%, 60%, 90%, 60%, and 30% acetone for 5 s each and then in distilled water. IF images were acquired with a Nikon AXR confocal or Carl Zeiss upright Apotome Axio Imager Z1/ZEN2 Core Imaging software. Cell quantification was done using NIH ImageJ software by cell counts in 10 separate 10X fields. Amira 5.4 software (VSG) was used for data capture and analysis. For Hypoxyprobe detection, whole‐cell lysates were prepared in a lysis buffer and analyzed by Western blotting with Hypoxyprobe‐1 antibody conjugated with FITC (Hypoxyprobe, 90531) and secondary anti‐FITC antibody (Cat. # 90532, 1:1000) conjugated with horseradish peroxidase (HRP). Anti‐β‐Actin (Abcam ab8226, 1:5000) antibody was used with anti‐mouse IgG‐HRP. Signal was quantified using the Odyssey CLx imaging system (LI‐COR).

### 2.4 DNA and RNA analyses

DNA was extracted as described (Gao et al., 2020). A quantitative real‐time PCR (qRT‐PCR) method for absolute telomere length was used as described (Gao et al., 2020; O'Callaghan & Fenech, 2011). RNA was extracted using the Trizol Reagent (Life Technologies, Cat. # 15596018). RNA sequencing (RNAseq) was performed by Novogene (Supplement) as reported (Gao et al., 2023). Complementary DNAs were generated using High Capacity cDNA Reverse Transcription Kit (Applied Biosystems, Cat. # 4368814). PCR reactions were performed on CFX96™ Real‐Time System C1000 Touch thermal cycler (Bio‐Rad) using Q‐PCR Master Mix (Gendepot, Cat. # Q5600‐005). Expression of mouse genes was normalized to *18S RNA*. Primer sequences are as follows: *Tert: forward 5′‐GGATTGCCACTGGCTCCG‐3′*, *reverse 5′‐TGCCTGACCTCCTCT TGTGAC‐3′; Cdkn2a*, *forward 5′‐AACATCTCAGGGCCGAAA‐3′*, *reverse 5′‐TGCGCTTGGAGTGATAGAAA‐3′; Pgc1a: forward 5′‐ GCACCAGAAAACAGCTCCAAG‐3′*, *reverse 5′‐CGTCAAACACAGCTTGACAGC‐3′; Nd1: forward 5′‐ CTAGCAGAAACAAACCGGGC‐3′*, *reverse 5′‐ CCGGCTGCGTATTCTACGTT‐3′; Hk2 forward 5′‐GCCAGCCTCTCCTGATTTTAGTGT‐3′*, *reverse 5′‐GGGAACACAAAAGACCTCTTCTGG‐3′; Hk1: forward 5′‐CATTGTCTCCTGCATCTCCGA*, *reverse 5′‐ ATTCCGCAATCTAGGCTCGTC‐3′; Ldh: forward 5′‐ ACGCAGACAAGGAGCAGTGGAA‐3′*, *reverse 5′‐ATGCTCTCAGCCAAGTCTGCCA‐3′; 18S: forward 5′‐ AAGTCCCTGCCCTTTGTACACA‐3′*, *reverse 5′‐GATCCGAGGGCCTCACTAAAC‐3′*. Fluorescence in situ hybridization (Telo‐FISH) was done on cells prepared by cytospin as described (Bellows et al., 2011). Briefly, after 4% paraformaldehyde fixation, 5 × 10^4^ cells/200 μL were loaded into cytology funnel (Biomedical polymers Inc, BMP‐Cyto‐S50) with slide and spun in Cytospin 4 (Thermal Scientific) at 800 rpm for 3 min. The filter was removed from the cytology funnel/slide. The cells in a flat layer on the slides were probed with a telomere‐specific TelC‐Cy3 (PNA Bio, F1002) as described (Gao et al., 2020). The slides, treated with blocking reagent (Roche), were preheated 5 min at 85°C followed by adding the TelC‐Cy3 probe (0.3 ng/mL) in hybridization buffer, incubation at 85°C for 10 min in a humidified chamber, and then hybridization at RT for 2 h. After 3 washes with PNA wash solution and PBS, samples counterstained with Hoechst were mounted in Fluoromount G medium.

### 2.5 Statistical analysis

Microsoft Excel and Graphpad Prism were used to graph data as mean ± SEM and to calculate *p*‐values using homoscedastic Student's *t*‐test. *p* < 0.05 was considered significant. All experiments were repeated at least twice with similar results.

## 3 RESULTS

### 3.1 Telomere attrition and senescence in adipose *Tert*‐KO EC

**FIGURE 1:**
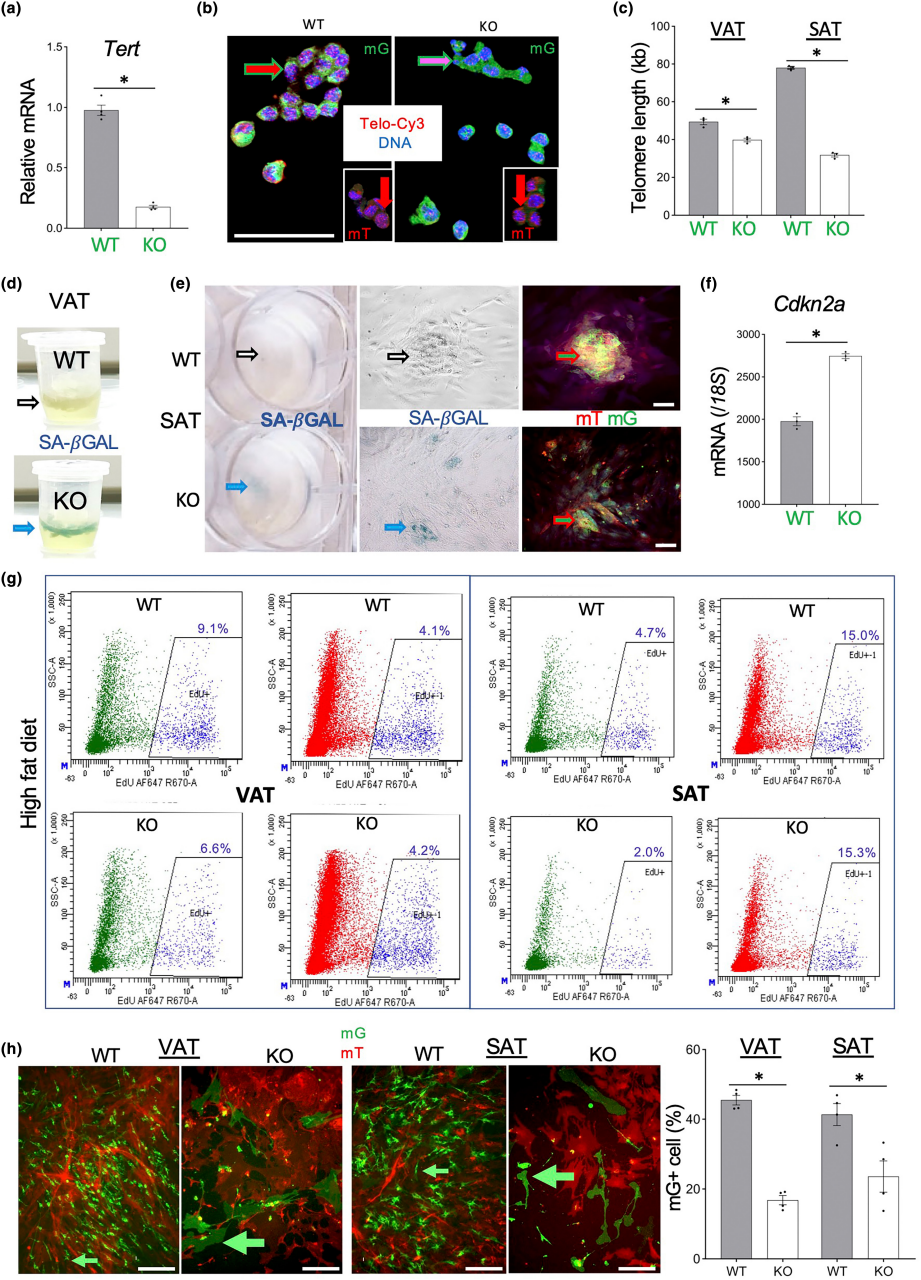
Endothelial cells (EC) *Tert* KO results in AT EC telomere attrition and senescence. (a) q‐RT‐PCR reveals *Tert* expression reduction in mG+ cells FACS‐sorted from SAT of EC‐*Tert*‐KO mice (8 months old). (b) Telo‐FISH reveals shorter telomeres (lower red TelC‐Cy3 signal) in mG+ cells (green outline arrow) from VAT of EC‐*Tert*‐KO mice (12 months old), whereas nuclear TelC‐Cy3 signal in mT+ cells (insets) is comparable. (c) q‐PCR on DNA from VAT and SAT mG+ lineage cells reveals shorter telomeres in HFD‐fed EC‐*Tert*‐KO mice at 8 months of age. Real‐time PCR data are normalized to data for a single copy gene. (d) SA‐β‐gal staining reveals senescence in VAT of EC‐*Tert*‐KO mice (8 months old). (e) SA‐β‐gal staining reveals senescence in cultured mG+ cells from SAT of EC‐*Tert*‐KO mice (8‐month‐old). Left: plate wells; middle: brightfield micrograph; right: fluorescence of the same area. EC colonies (mG+) are intermixed with stromal cells (mT+), which results in yellow color. (f) q‐RT‐PCR reveals higher *Cdkn2a* expression (normalized to *18S* RNA) in mG+ cells FACS‐sorted from SAT of EC‐*Tert*‐KO mice (8 months old). (g) Flow cytometry on SVF recovered 3 days after EdU injection comparing incorporation into mG+ and mT+ cells in VAT, and SAT of HFD‐fed mice at 9 months of age. EdU fluorescence: 647 nm channel, side scatter is used for separation. (h) Primary culture of SVC from VAT and SAT of 7‐month‐old mice 2 days after plating at identical density. Note reduced proliferation and the large size of mG+ cells from EC‐*Tert*‐KO mice. For all data, shown are mean ± SEM (error bars). *p < 0.05 (two‐sided Student's *t*‐test). Scale bar = 100 μm.

To generate mice with *Tert* KO in EC, we crossed *Tert* ^*fl/fl*^ mice (Liu et al., 2015) with mice expressing *Cre* under the control of EC‐specific *Tie2e* promoter (Kano et al., 2003). All mice also carried the *mTmG* reporter (Muzumdar et al., 2007) to enable identification of EC as cells expressing membrane GFP (mG) among cells expressing membrane Tomato (mT). *Tie2e‐cre*; *Tert* ^*fl/fl*^; *mTmG* (KO) and *Tie2e‐cre*; *Tert* ^*+/+*^; *mTmG* (WT) littermates were identified by PCR genotyping and by green fluorescence of the vasculature in ears. EC‐*Tert*‐KO mice did not display developmental abnormalities. Over a year of monitoring, there were no notable signs of accelerated aging or pathology. Adipose tissue EC incapacitation and angiogenesis suppression has been reported to suppress obesity development (Kolonin et al., 2004; Rupnick et al., 2002). Based on that, we reasoned that white adipose tissue (WAT) expanding in obesity is most likely to reveal exhaustion of EC. To test this, mice were fed high‐fat diet (HFD) from 1 to 8 months of age. Cells isolated from subcutaneous AT (SAT) were subjected to FACS sorting to isolate mG+ for mRNA extraction. Validating knockout efficiency, analysis of RNAseq reported recently (Gao et al., 2023) demonstrated *Tert* downregulation (1300‐fold) to be among the most pronounced, which was linked with changes of expression in genes engaged in DNA repair and other cellular pathways (Figure S1a,b). By using qRT‐PCR, we confirmed that expression of *Tert* was dramatically lower in *Tert*‐KO EC than in WT EC (Figure 1a). Telomere length was analyzed by fluorescent in situ hybridization (FISH) with a telomere probe in mixed stromal/vascular cells (SVC) from SAT. Telo‐FISH revealed shorter telomeres in EC of KO mice, whereas telomere length in mT+ cells was comparable in EC and KO mice (Figure 1b). We confirmed this by a quantitative PCR (qPCR) assay that measures the rate of telomeric DNA amplification relative to a single copy gene (O'Callaghan & Fenech, 2011), hence determining the relative quantities of the telomeric hexamer repeats. Using this approach, expedited (compared to WT mice) telomere attrition was evident in both SAT and visceral AT (VAT) mG+ cells FACS‐sorted from KO mice raised on HFD (Figure 1c).

We then investigated if *Tert* KO in EC led to their expedited senescence by using conventional assays. Senescence‐associated β‐galactosidase (SA‐β‐gal) activity was higher in both AT of KO mice compared to WT mice (Figure 1d). Analysis of adherent SVC isolated from AT confirmed that SA‐β‐gal activity was detected in mG+ EC (Figure 1e). The effect of *Tert* KO was also analyzed by transcriptomic analysis of FACS‐sorted mG+ cells. Comparative Ingenuity Pathway Analysis (IPA) of RNAseq data demonstrated induction of key pathways mediating senescence in EC from AT of KO mice (Figure S2a). Transcripts induced in *Tert*‐KO EC included *Cdkn1a*, *Cdkn2a*, *Cdkn2b*, *Cdkn2d*, *Atm*, *Chek2*, *Tp53*, *as well as SASP cytokines Il6*, *Il8*, *Tgfb*, *and Ifn1*. Expression of a key senescence effector, *Cdkn2a*, in FACS‐sorted mG+ SAT cells was confirmed by qRT‐PCR to be significantly (*p* = 0.018) higher in KO mice than in WT mice (Figure 1f). Interestingly, these manifestations of senescence were observed even for mice fed chow, indicating that excessive replicative exhaustion by HFD‐induced obesity is not necessary for senescence induction.

Using 5‐ethynyl‐2′‐deoxyuridine (EdU), a fluorescent nucleotide analog incorporating into replicating DNA, we compared the effect of *Tert* KO on EC proliferation. EdU was injected into mice, and FACS on the SVC was used to quantify EdU‐positive mG+ and mT+ cells. In both SAT and VAT, proliferation frequency in mT+ cells was comparable in KO and WT mice. In contrast, it was 1.4‐fold (VAT) and 2.35‐fold (SAT) lower in EC of KO mice raised on HFD, compared to WT controls raised on HFD (Figure 1g). EC cell proliferation was also reduced in AT of KO mice raised on regular chow, although the difference for SAT was only 1.5x lower than in WT EC (data not shown). To analyze EC proliferation ex vivo, we cultured SVC from SAT and VAT. Before the primary cells were passaged, phenotypic differences were already apparent between WT and KO cells (Figure 1h). EC from KO mice were markedly large, which is characteristic of senescence, and failed to proliferate, unlike WT EC. These data demonstrate that *Tert* KO in EC leads to their replicative senescence, onset of which is aggravated by HFD feeding.

### 3.2 Dysfunction and mis‐differentiation of adipose *Tert*‐KO EC

**FIGURE 2:**
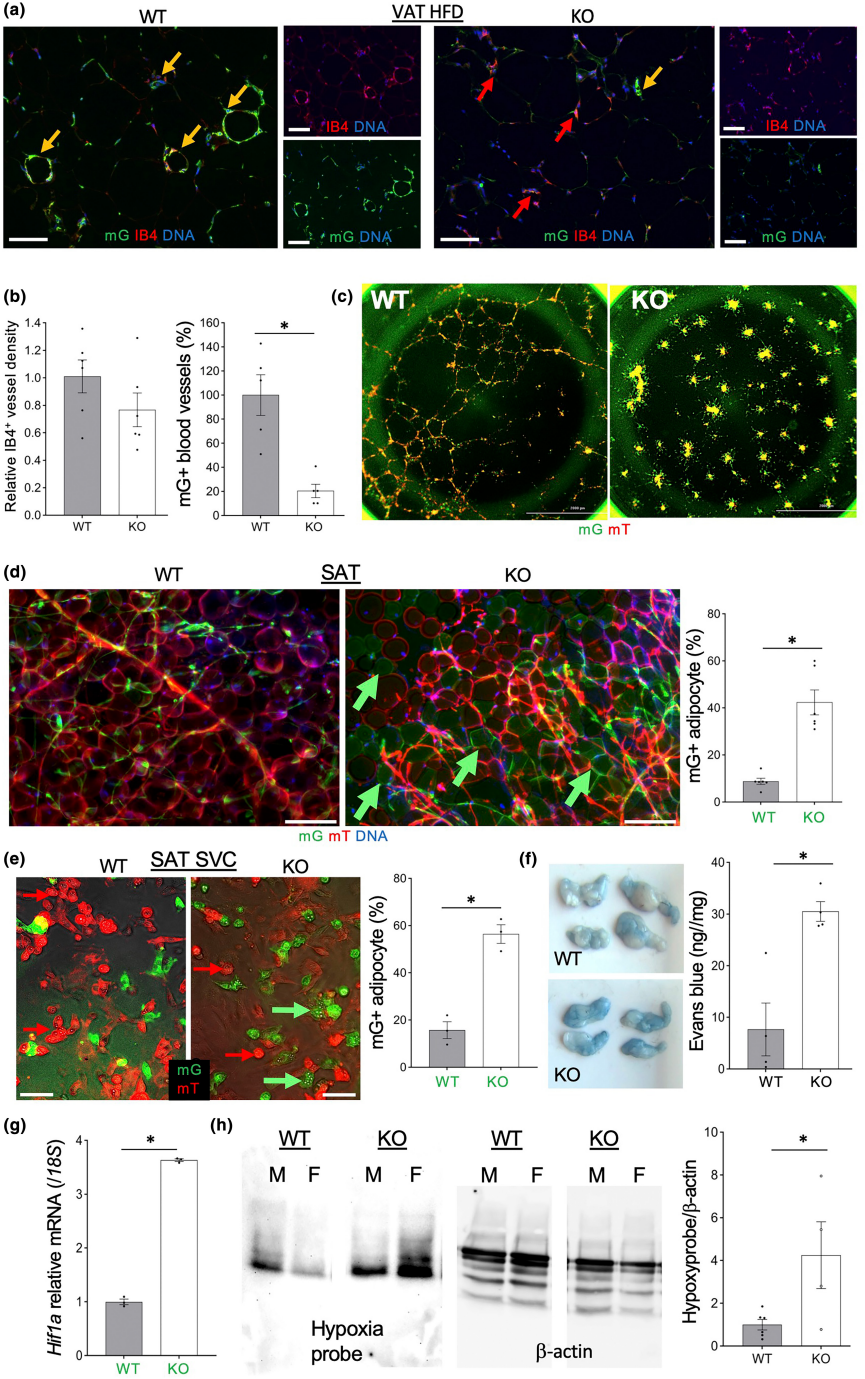
*Tert* KO causes endothelial cells (EC) dysfunction, mis‐differentiation, and vessel leakiness. (a) Anti‐GFP IF analysis in tissue sections, with IB4 counterstaining EC, reveals a lack of mG+ cells in blood vessels in VAT of EC‐*Tert*‐KO mice fed HFD (9 months). (b) Data quantification from (a) based on IB4 binding and mG expression in the vasculature. (c) Self‐organization of SAT mG+/mT+ SVC into stromal‐vascular networks showing a defect of *Tert*‐KO EC. (d) Whole mount of SAT showing increased frequency of EC‐derived adipocytes in AT of EC‐*Tert*‐KO mice. Graph: Data quantification. (e) Primary culture of SVC from SAT after adipogenesis induction showing increased differentiation of *Tert*‐KO mG+ cells into adipocytes containing lipid droplets. Graph: Data quantification. (f) VAT 0.5 h after intravenous injection of Evans blue and subsequent systemic perfusion showing increased dye retention in EC‐*Tert*‐KO mice. Graph: Dye retention quantification. (g) q‐RT‐PCR reveals higher *Hif1a* expression (normalized to *18S* RNA) in mG+ cells FACS‐sorted from SAT of EC‐*Tert*‐KO mice (8 months old). (h) Extracts from VAT 0.5 h after injection of hypoxyprobe analyzed by PAGE. β‐Actin immunoblotting: Loading control. Note increased hypoxyprobe retention in VAT of EC‐*Tert*‐KO males (M) and females (F). Graph: Data quantification: Mean ± SEM. For all data, shown are mean ± SEM (error bars). *p < 0.05 (two‐sided Student's *t*‐test). Scale bar = 50 μm.

To determine if *Tert* KO affects the vasculature, we analyzed AT sections. Because paraffin embedding inactivates mG and mT fluorescence, we performed IF to detect mG expression. Analysis of SAT and VAT sections from mice raised on chow did not reveal notable differences in the vasculature and in the tissue distribution of mG+ cells (data not shown). However, after prolonged HFD feeding, there were marked differences revealed by anti‐GFP antibody. Counterstaining with isolectin B4 (IB4), binding to EC, showed that vascular density and morphology were similar in AT of KO and WT mice (Figure 2a). Quantification of microvascular density based on IB4 binding also did not reveal a significant difference (Figure 2b). However, IF revealed that mG+ cells contributed to the vasculature much less in KO AT than in WT AT (Figure 2b). This indicates that HFD feeding leads to the exhaustion of the Tie2e + lineage in KO mice. Instead, cells of a different (*Tert*+) lineage are recruited to compose the vasculature. *Tert*‐KO EC were also found to be dysfunctional ex vivo. When SVC were subjected to 3D vascularization assay (Min et al., 2016), WT cells formed endothelial networks whereas KO cells did not (Figure 2c).

To determine the fate of *Tert*‐KO cells that fail to contribute to the vasculature, we quantified their contribution to the adipogenic lineage. Consistent with reports indicating that EC occasionally give rise to adipocytes (Gupta et al., 2012; Tran et al., 2012), we detected rare mG+ adipocytes in SAT of WT mice (Figure 2d). Similar trends were observed for VAT and BAT (data not shown). Significantly higher frequency of mG+ adipocytes was observed in SAT from KO mice compared to control littermates (Figure 2d). Predisposition of *Tert*‐KO EC to adipocyte differentiation was also observed in primary culture of SVC induced to undergo adipogenesis. While mainly mT+, but not mG+, cells formed lipid‐laden adipocytes in WT SVC, almost all *Tert*‐KO mG+ cells accumulated lipid droplets to a comparable level (Figure 2e). This indicates that *Tert*‐KO EC undergo an increased conversion to adipogenic lineage. Importantly, this was observed even for cells derived from young mice, suggesting that this mis‐differentiation occurs independently of telomere attrition observed in 8 month‐old HFD‐fed mice. Mis‐differentiation of Tert‐KO EC was also detectable in SVC isolated from AT and labeled with IB4 in cell culture (Figure S1c).

### 3.3 *Tert* KO in EC results in vascular leakiness and widespread hypoxic response

Induction of pathways hallmarking EC dysfunction was confirmed in the transcriptome of FACS‐sorted mG+ cells demonstrated the in *Tert*‐KO EC subjected to comparative IPA (Figure S2b). This was apparent for EC from SAT and VAT of mice raised on either HFD or chow, further indicating that cell dysfunction arises irrespective of replicative exhaustion. To analyze vasculature functionality in vivo, we injected Evans blue dye via tail vein into WT and KO mice raised on chow. Visual assessment of the rate of dye distribution among the mouse organs did not reveal a limitation of perfusion in KO mice. After perfusing the mouse through the heart, we analyzed dye retention. SAT and VAT pads of KO mice were notably more colored, compared to WT mice (Figure 2f). This difference was confirmed to be statistically significant upon calorimetric Evans blue quantification in tissue suspension (Figure 2f). This indicates that *Tert* KO in EC results in increased vascular leakiness. We also assessed vascular perfusion by injecting red fluorescent microspheres via tail vein and comparing their biodistribution in organs of WT and KO mice (Figure S1d). Overall, microsphere signal was comparable in AT of WT and KO mice, indicating that Tert KO microvasculature is well perfused. Interestingly, while in WT mice microspheres were detected mainly luminally, in KO mice their accumulation was apparent in the parenchyma, confirming vasculature leakiness.

Because increased vascular permeability is linked with hypoxia (Ogawa et al., 1992; Stenmark et al., 2006), we looked for the evidence of its activation in the transcriptome of *Tert*‐KO EC. Indeed, top canonical pathways found to be upregulated in *Tert*‐KO EC converged on genes implicated in hypoxia, including hypoxia inducible factor *Hif1a* (Figure S2c). Expression of *Hif1a* was confirmed by q‐RT‐PCR to be significantly (*p* < 0.0001) higher in EC from KO chow‐fed mice, compared to EC from WT chow‐fed mice (Figure 2g). To test if this is a reflection of the actual tissue hypoxia, we injected mice with Hypoxyprobe and analyzed its tissue accumulation. In both males and female mice, VAT Hypoxyprobe accumulation was found to be significantly higher in KO mice than in WT mice (Figure 2h). This demonstrates that *Tert* KO in EC predisposes to increased tissue hypoxia.

We then investigated if this effect on AT physiology is contributed to by changes in cells other than EC. For this, AT from KO and WT male mice fed with HFD from 2 to 7 months of age was subjected to single‐cell RNA sequencing (scRNAseq). Using cluster assignments described previously (Gao et al., 2020), we identified adipose stromal cells (ASC), smooth muscle cells (SMC), and the leukocyte subpopulations as recently reported (Gao et al., 2023). For non‐endothelial cells, *Tert* expression was comparable between KO and WT mice. For example, there was no *Tert* mRNA difference between ASC from KO VAT versus WT VAT (*p* = 0.4) or in B‐cells from KO SAT versus WT SAT (*p* = 0.8). However, there was a global effect on non‐endothelial cells. SAT of EC‐*Tert*‐KO mice had an increase in T‐cells while the presence of B‐cells was reduced. IPA analysis of gene expression revealed induction of a range of pathways in ASC of KO mice (Figure S3a). Interestingly, genes implicated in mitochondrial dysfunction were induced the most. Other pathways indicating metabolic stress were also activated, including CLEAR signaling, sirtuin signaling, and autophagy. The HIF1a pathway was also upregulated consistent with the induction of fibrosis pathway activation also detected. Specific analysis of the hypoxia pathway response in ASC revealed activation of a vast number of genes acting upstream and downstream of HIF1a (Figure S3b). They included *Tp53*, also activated during the induction of senescence, which prompted us to assess this pathway in the ASC. Interestingly, the senescence gene signature was also activated in ASC, including *Chek1*, *Cdkn2a*, *Cdkn2b*, *Cdkn2d*, as well *Cxcl8*, *Ifn1* and other SASP genes (Figure S3c). Similar responses were detected in SMCs and leukocytes (data not shown). These results indicate that *Tert* inactivation in EC results in hypoxia and a widespread bystander stress on other cells of AT.

### 3.4 Reduced adiposity and abnormal metabolism of EC‐*Tert*‐KO mice fed HFD

**FIGURE 3:**
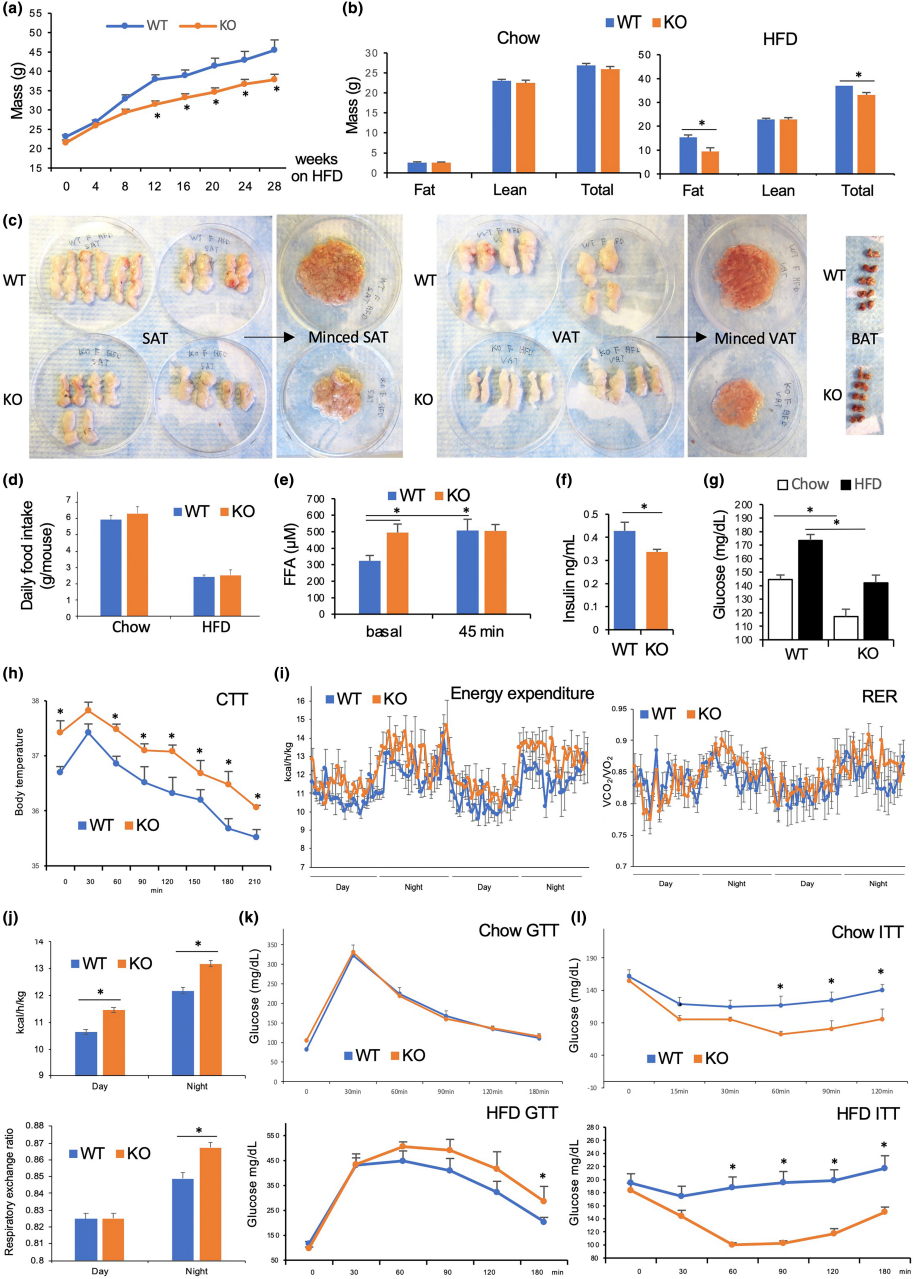
High calorie diet reveals EC‐*Tert*‐KO WAT abnormality and metabolism dysfunction. (a) The time course of body weight change in mice fed HFD. (b) Body composition measured in males fed chow or HFD for 3‐months. (c) AT resected depots from female mice, whole and minced, show reduced SAT and VAT adiposity of EC *Tert* KO. (d) Daily food consumption of mice on normal chow and HFD. (e) Plasma‐free fatty acid concentration increase after isoproterenol administration, not observed in EC‐*Tert*‐KO mice. (f) Plasma insulin concentration is lower in EC‐*Tert*‐KO mice. (g) Plasma glucose concentration in mice fed chow or HFD, lower in EC‐*Tert*‐KO mice. (h) Cold tolerance test: body temperature measured after placement at 4°C. (i) Calorimetric measurement of energy expenditure and respiratory exchange ratio (RER), calculated as VCO_2_/VO_2_, over 2 days. (j) Mean values for all timepoints in (i). (k) Glucose tolerance test (GTT) after 6 months of chow and HFD feeding. (l) Insulin tolerance test (ITT) after 6 months of chow and HFD feeding. For all data, shown are mean ± SEM (error bars). *p < 0.05 (two‐sided Student's *t*‐test). Scale bar = 50 μm.

To determine how EC *Tert* KO and AT cell dysfunction affects physiology, we compared metabolism in WT and KO mice raised on chow or HFD. While there was no difference in body weight for KO vs WT mice raised on chow, HFD‐fed KO mice gained significantly less weight than their WT littermates (Figure 3a). Echo MRI analysis demonstrated that this was specifically due less fat mass accumulation in KO mice (Figure 3b). Resection of AT depots demonstrated that the volume of both SAT and VAT was lower in KO mice, whereas BAT volume was comparable (Figure 3c). Consumption of either diet was not statistically different between KO and WT mice, hence ruling out caloric intake as a possible explanation (Figure 3d). To investigate a potential reason for reduced adiposity of KO mice, we analyzed circulating lipids. Concentration of free fatty acids (FFAs) was found to be significantly higher in plasma of KO mice compared to WT (Figure 3e). This suggested that WAT in KO mice is inefficient in depositing dietary lipids, which could account for reduced adiposity. Upon lipolysis induction by β‐adrenergic agonist isoproterenol, FFAs in the circulation significantly increased in WT mice but was not further increased in KO mice (Figure 3e). This suggested that WAT in KO mice has an increased baseline level of FA mobilization, which could also contribute to reduced adiposity.

We then analyzed glucose metabolism changes upon EC *Tert* KO. Steady state circulating insulin levels were significantly lower in KO littermates (Figure 3f). Non‐fasting glucose levels were increased by prolonged HFD feeding in both WT and KO mice (Figure 3g). On either diet, non‐fasting glucose levels were significantly lower in KO mice compared to WT littermates (Figure 3g). To determine if the observed lipid/glucose disbalance in circulation of KO mice is linked with thermogenic AT dysfunction, we performed the cold tolerance test. KO mice were found to maintain body temperature at least as well as WT littermates. Interestingly, KO mice fed HFD were found to have chronically higher body temperature than WT littermates (Figure 3h). Transcriptomic analysis of *Tert*‐KO EC identified increased expression of genes coding for pyrogenic factors, including leukotriene 4, as well as of prostaglandins *Pgf2a*, *Pgd2*, and *Pgi2*. Consistent with chronic low‐grade fever detected, indirect calorimetry analysis revealed that KO mice had higher energy expenditure, as determined based on oxygen consumption (Figure 3i). The difference was statistically significant both at nighttime and daytime (Figure 3i). The respiratory exchange ratio (RER) calculation revealed that KO males used more glucose as a metabolic substrate, compared to control mice (Figure 3i), which was statistically significant at night (Figure 3j). Glucose administration into fasted mice did not reveal a difference between KO and WT littermates (Figure 3k). However, upon HFD feeding, KO mice had a slightly higher glucose tolerance than WT littermates (Figure 3k). Insulin administration into fasted mice indicated that KO males were significantly more insulin‐sensitive irrespective of the diet (Figure 3l). Together, these metabolic data indicate an increase in glucose metabolism in mice lacking *Tert* in EC.

### 3.5 EC‐*Tert*‐KO mice have brain dysfunction despite a lack of telomere attrition

**FIGURE 4:**
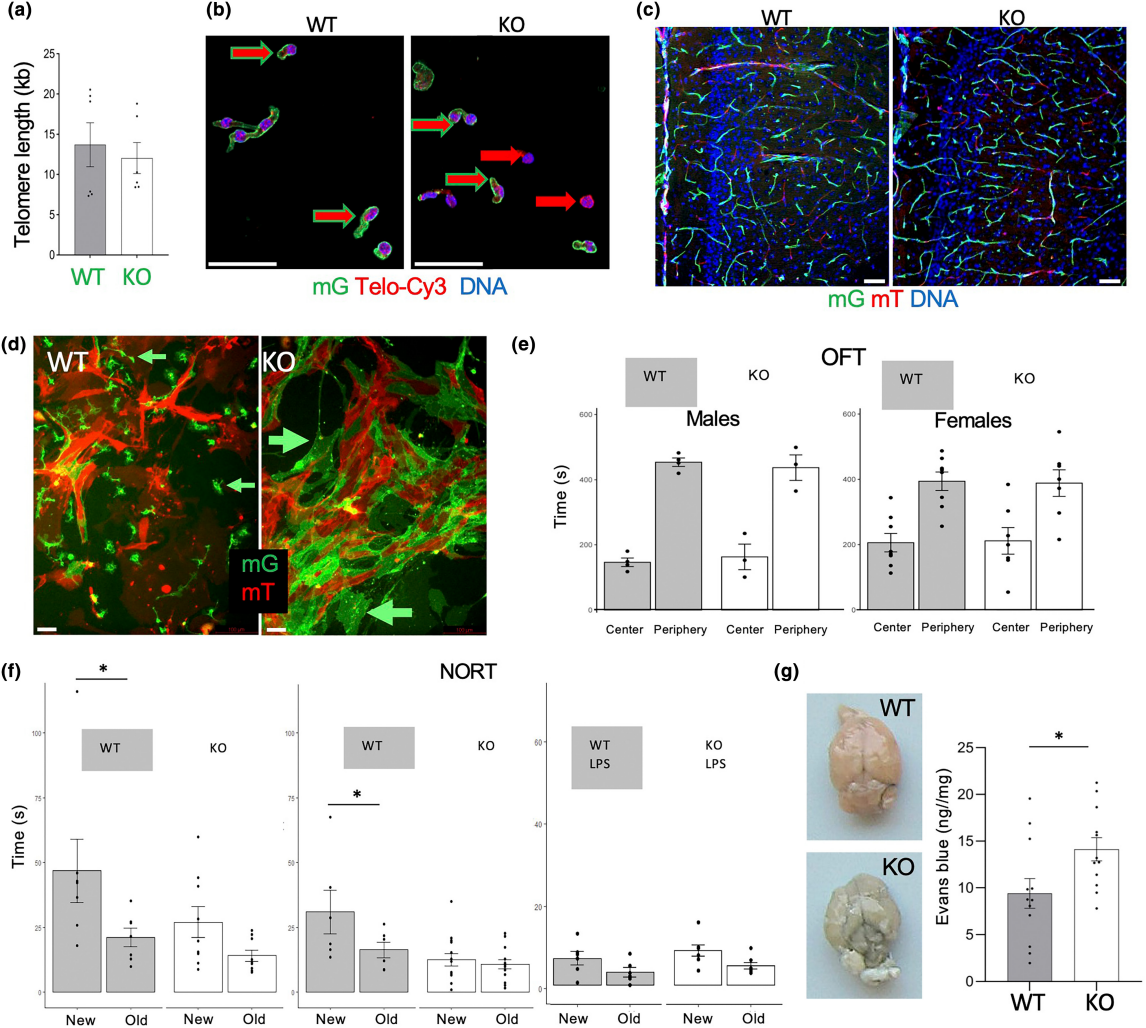
*Tert* KO impairs brain ECs and cognitive function without telomere attrition. (a) q‐PCR on DNA from brain mG+ lineage cells of EC‐*Tert*‐KO mice at 10 months of age. Real‐time PCR data are normalized to data for a single copy gene. (b) Telo‐FISH reveals comparable telomere length (red TelC‐Cy3 signal) in mG+ cells (green outline arrow) from EC‐*Tert*‐KO and WT mice. (c) mG and mT fluorescence in hypothalamus sections reveals normal vasculature in EC‐*Tert*‐KO mice. (d) Primary culture of cells from the brains 2 days after plating at identical density. Note reduced proliferation and larger size of mG+ EC‐*Tert*‐KO cells. (e) Open field test does not reveal behavioral abnormality in EC‐*Tert*‐KO mice. (f) Novel object recognition test (NORT) reveals memory impairment in EC‐*Tert*‐KO males and females. Lipopolysaccharide (LPS) injection, impairing memory, was used as a positive control. (g) Brains 0.5 h after tail vein injection of Evans blue and subsequent systemic perfusion showing increased dye retention in EC‐*Tert*‐KO mice. Graph: calorimetric Evans blue quantification. For all data, shown are mean ± SEM (error bars). *p < 0.05 (two‐sided Student's *t*‐test). Scale bar = 50 μm.

Chronic low‐grade hyperthermia observed in EC‐*Tert*‐KO mice (Figure 3h), suggested an effect on thermoregulation, which is controlled by the CNS. Analysis of mG+ cells isolated by FACS from the brain of 8‐month‐old mice by q‐PCR did not detect a difference in telomere length between KO and WT mice (Figure 4a). No difference was also detected between brain WT and *Tert*‐KO EC by telo‐FISH (Figure 4b). Density and morphology of blood vessels analyzed in tissue sections of the hypothalamus were also comparable in KO and WT mice (Figure 4c). However, EC from the brains of *Tert*‐EC‐KO mice had an obvious senescence phenotype when adherent in cell culture (Figure 4d).

To test if EC *Tert* loss and senescence affects behavior, we performed a number of conventional tests. All aspects of diurnal locomotor activity appeared normal in KO mice, including inter‐daily stability and intradaily variability, circadian amplitude, and MESOR, as acquired by infrared sensor monitoring of home cage locomotion (data not shown). Because disruption of the circadian rhythm has been shown to cause endothelial damage (Carreras et al., 2014), we tested whether circadian disruption of the central pacemaker (the suprachiasmatic nucleus) would enhance the effect of EC *Tert* loss on CNS function. However, housing mice in constant light also did not reveal any behavioral changes beyond those seen for WT littermates. No signs of stress or anxiety were detected in the open field test (OFT) for either males or females (Figure 4e). To test cognitive function, we used the novel object recognition test (NORT) (Lissner et al., 2021), which is a test for long term memory consolidation. As a positive control for the effects of vascular damage on cognition, we used LPS, which activates adhesion molecules and chemokines, which leads to increased vascular permeability and neuroinflammation (Batista et al., 2019). Consistent with previously reported effects of LPS on memory in mice (Engler‐Chiurazzi et al., 2023), novel object recognition observed for WT mice was impaired by LPS pre‐treatment (Figure 4f). In this assay, both male and female *Tert*‐EC‐KO mice displayed a reduced ability to recognize novel objects, compared to WT littermates (Figure 4f). This impairment was comparable to that caused by LPS in WT mice, indicating the importance of endothelial *Tert* for cognitive function.

To determine if the phenotype observed is due to the loss of vasculature functionality, we analyzed the brains of mice injected with Evans blue dye via tail vein and then perfused through the heart (Figure 2f). Consistent with the notion that Evans blue normally does not cross the BBB (Serghides et al., 2011), there was no dye retention observed in WT mice. In contrast, the brain of KO mice was visibly blue (Figure 4g). This difference was confirmed to be statistically significant upon calorimetric Evans blue quantification in tissue suspension (Figure 4g). Brain sections through hypothalamus confirmed Evans blue far‐red fluorescence in the preoptic area, which controls thermoregulation (data not shown). These data indicate increased vascular leakiness in the brain of EC‐*Tert*‐KO mice.

### 3.6 *Tert* KO impairs muscle ECs and endurance without telomere attrition

**FIGURE 5:**
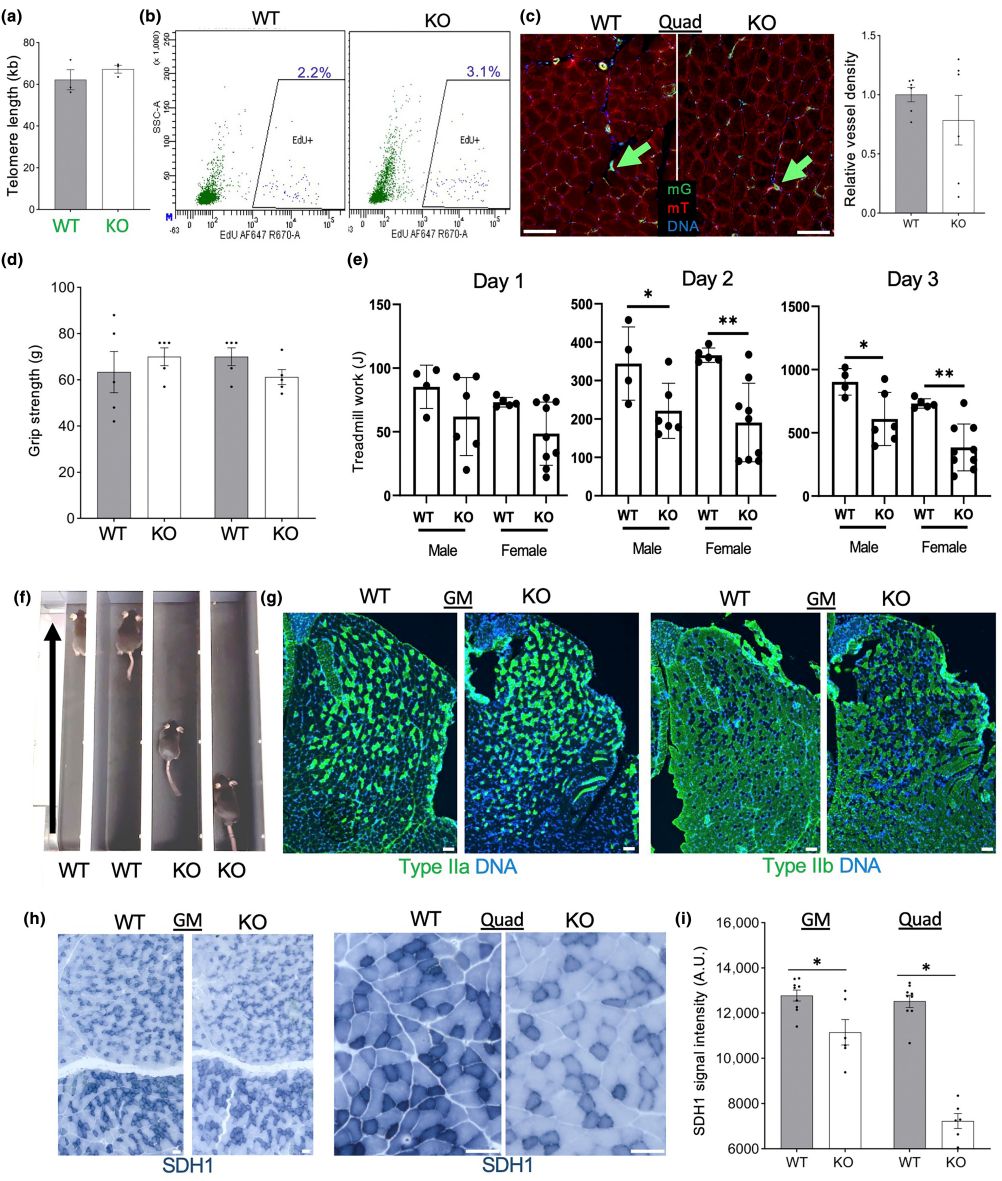
*Tert* KO impairs muscle ECs and function without telomere attrition. (a) q‐PCR on DNA from combined quadriceps (Quad) and gastrocnemius (GM) muscle mG+ lineage cells of EC‐*Tert*‐KO mice at 10 months of age. Real‐time PCR data are normalized to data for a single copy gene. (b) Flow cytometry on hindlimb skeletal muscle cells recovered 3 days after EdU injection comparing incorporation into mG+ and mT+ cells. (c) Fluorescence analysis of mG+ and mT+ cells in Quad muscle. Graph: data quantification based on vascular mG expression. (d) Grip strength, measured for forelimb and hindlimb of EC‐*Tert*‐KO and WT littermates (*N* = 5). (e) Quantification of data from e for 10 daily runs of *N* = 5 WT and *N* = 5 EC‐*Tert*‐KO mice, showing reduced physical endurance of EC‐*Tert*‐KO mice, reflected in Joules of work performed (weight (kg) × speed (m/min) × time (min) × incline (degree) × 9.8 m/s^2^). (f) A snapshot of WT and EC‐*Tert*‐KO littermates running on a treadmill (arrows: direction) illustrating reduced fatigue resistance capacity in EC‐*Tert*‐KO mice. (g) IF analysis of muscle fiber types in GM muscle. (h) Immunohistochemistry on GM and Quad reveals decreased succinate dehydrogenase (SDH1) expression in the muscle fibers of EC‐*Tert*‐KO mice. (i) SDH1 expression quantification from (h). For all data, shown are mean ± SEM (error bars). *p < 0.05 (two‐sided Student's *t*‐test). Scale bar = 50 μm.

A decrease in muscle capacity is another hallmark of aging. To test if *Tert* KO predisposes EC in the skeletal muscle to telomere attrition, we isolated mG+ cells by FACS from quadriceps (Quad) and gastrocnemius (GM) of 8‐month‐old mice and measured telomere length. No significant difference was detected by q‐PCR between KO and WT mice (Figure 5a). By injecting EdU, we compared the effect of *Tert* KO on muscle EC proliferation. FACS analysis of hindlimb muscle cells did not detect a reduction in the frequency of EdU + mG+ cells in KO mice compared to WT mice (Figure 5b). Density and morphology of blood vessels analyzed in tissue sections of Quad or GM muscles were also comparable in KO and WT mice (Figure 5c). To determine if *Tert* loss in EC affects muscle function, we measured grip strength. Both forelimb and hindlimb strength were as at least as high in KO mice as in WT littermates (Figure 5d), indicating that the function of fast‐twitch muscles is not jeopardized by EC *Tert* KO. To measure muscle endurance, mice were challenged by treadmill running at the regimen that leads to exhaustion within 10 min (Figure 5e,f). On Day 1, there was a trend for KO mice maintaining running for lower period of time than WT littermates; subsequent Days 2 and 3, this difference had become significant for both males and females (Figure 5e,f). Decreased fatigue resistance in KO mice suggested an impairment of in the function of slow‐twitch muscle fibers. However, IF analysis did not reveal a decrease in the frequency of Type IIa oxidative muscle fiber types in Quad or GM muscles (Figure 5g). On the contrary, Type IIb fibers appeared to be underrepresented (Figure 5g). Because oxidative fibers rely on the function of mitochondria, we analyzed the activity of succinate dehydrogenase (SDH1), a marker for mitochondria oxidative capacity (Figure 5h). Analysis of tissue sections revealed a significantly lower SDH1 activity in both Quad and GM (difference less prominent) of KO mice (Figure 5i). These data indicate that the function of oxidative muscle fibers is reduced upon TERT inactivation in EC.

### 3.7 Reduced mitochondrial function and increased glycolysis in *Tert*‐KO EC

**FIGURE 6:**
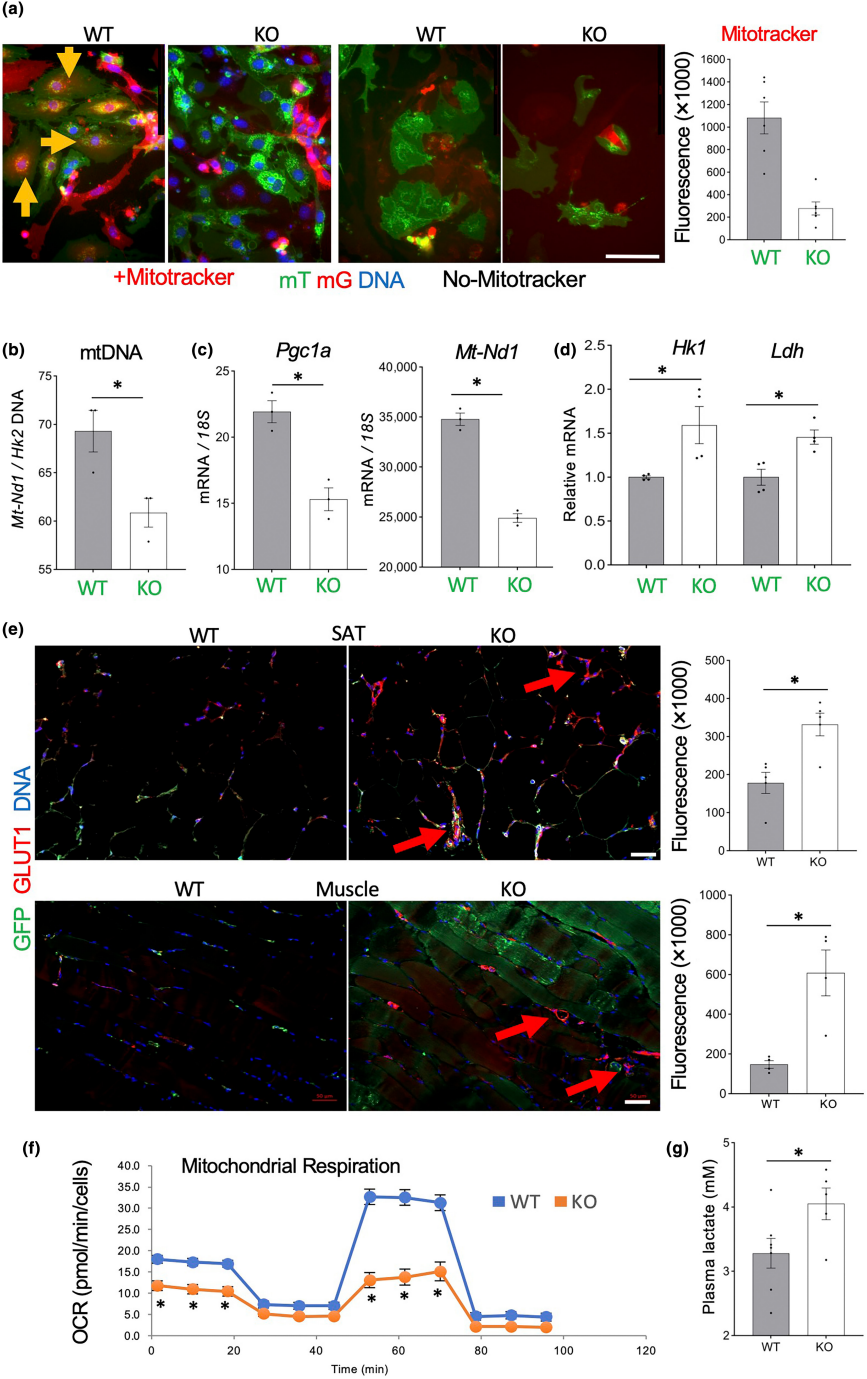
*Tert*‐KO endothelial cells (EC) have reduced mitochondrial function and increased glycolysis. (a) Primary culture of mixed mT/mG SVC from SAT of 12‐month‐old mice 2 days after plating at identical density stained with Mitotracker (red). Note that intracellular Mitotracker signal (arrow) is reduced in mG+ *Tert*‐KO EC. Graph: data quantification. (b) Mitochondrial DNA content, based on the ratio of *ND1* to *HK2* gene expression measured by q‐RT‐PCR in mG+ cells FACS‐sorted from SAT of EC‐*Tert*‐KO mice (8 months old). (c) q‐RT‐PCR reveals lower expression of mitochondrial respiration genes *PGC1a* (normalized to *18S* RNA). (d) q‐RT‐PCR reveals higher expression of glycolysis effectors *HK1* (*p* = 0.03) and *LDH* (*p* = 0.009) (normalized to *18S* RNA). (e) IF analysis of GLUT1 expression showing its induction colocalized with mG+ blood vessels in SAT and skeletal muscle sections of EC‐*Tert*‐KO mice. Graphs: data quantification. (f) Seahorse XF Cell Mito Stress Assay revealing decreased basal and induced oxygen consumption rate (OCR). (g) Increased lactate concentration in plasma of EC‐*Tert*‐KO mice. For all data, shown are mean ± SEM (error bars). *p < 0.05 (two‐sided Student's *t*‐test). Scale bar = 50 μm.

Reduced function of oxidative muscles, despite the lack of an apparent reduction in the numbers of oxidative fibers expressing SDH1 (Figure 5), suggested that their function is limited by mitochondria. To test this, we stained cultured SVC with Mitotracker‐red identifying active mitochondria. In this experiment, Mitotracker staining was apparent in WT mG+ EC, while it was obscured by mT+ staining in other cells (Figure 6a). Strikingly, significantly less Mitotracker binding was detected in mG+ cells from EC‐*Tert*‐KO mice (Figure 6a). We also quantified mitochondrial DNA by qPCR assay measuring the ratio of mitochondrial DNA amplification relative to autosomal DNA (*mt‐Nd1 / Hk2*). This revealed that *Tert*‐KO EC have significantly lower total mitochondria numbers (Figure 6b, *p* = 0.032). Consistent with that, expression of a key mitochondrial biogenesis gene, *Pgc1a*, was significantly lower in *Tert*‐KO EC (Figure 6c). There was also a significant reduction in the expression of NADH dehydrogenase subunit 1 (*Mt‐Nd1*), the key effector of mitochondrial electron transport. Reduced expression of protein‐coding mitochondrial genes in KO EC compared to WT EC was also evident from RNAseq of SAT GFP+ cells: mt‐Atp8 reduced 4.6‐fold (*p* = 0.007); mt‐Atp6 reduced 2.4‐fold (*p* = 0.003); mt‐Co2 reduced 2.4‐fold (*p* = 0.003); mt‐Nd1 reduced 2.1‐fold (*p* = 0.01); and mt‐Cytb reduced 2.0‐fold (*p* = 0.01). These data demonstrate that *Tert*‐KO EC have mitochondrial inadequacy. Downregulation of a range of genes engaged in the TCA cycle were also observed by Ingenuity Pathway analysis (data not shown).

These findings combined with increased glucose consumption observed for EC‐*Tert*‐KO mice (Figure 3) suggested their shift to glycolytic metabolism. Consistent with this, expression of genes coding for key glycolysis enzymes, Hexokinase 1 (*Hk1*), and lactate dehydrogenase (*Ldh*) was significantly higher in *Tert*‐KO EC than in WT EC (Figure 6d). Upregulation of a range of genes implicated in glycolysis were also observed by Ingenuity Pathway Analysis (data not shown). To confirm this at the level of protein expression, we performed IF with antibodies against GLUT1, the glucose transporter activated in glycolysis (Cho et al., 2017). GLUT1 expression in the vasculature, revealed by GFP IF, was found to be significantly higher in EC‐*Tert*‐KO in both AT and skeletal muscle (Figure 6e). To directly assess metabolism of EC, we performed the Seahorse XF Cell Mito Stress Assay on cultured SVC. Lower basal and induced oxygen consumption rate (OCR) revealed a defect in mitochondrial respiration of *Tert*‐KO EC (Figure 6f). Finally, analysis of mouse plasma demonstrated higher lactate concentration in KO mice, compared to WT mice (Figure 6g). Combined, our data demonstrate that *Tert* KO in EC results in their mitochondrial dysfunction leading to a metabolic shift from oxidative phosphorylation to glycolysis.

## 4 DISCUSSION

Progress in ameliorating aging‐associated cardiovascular and degenerative disease remains limited, at least in part, because current clinical approaches do not adequately take into account aging as a driver (Fossel et al., 2022). The causes of EC senescence have remained incompletely understood; however, inactivation of TERT in postnatal human development appears to play a pivotal role (Chakravarti, LaBella, & DePinho, 2021). Here, we used mice, which continue to express *Tert* in progenitor cells in the adulthood (Prowse & Greider, 1995), as a model to test the role of TERT in EC. Global *Tert* KO mice in G1 generation analyzed at 4 months of age were found to have largely intact telomeres and no overt phenotype in tissues analyzed, yet, repression of genes regulating oxidative phosphorylation and reduced mitochondrial content was already observed in liver, heart, and hematopoietic cells (Sahin et al., 2011). Consistent with and extending these observations, our data indicate that endothelium‐specific *Tert* inactivation causes EC senescence in part through a telomere‐independent mechanism undermining mitochondrial respiration, increasing vascular permeability, and causing tissue hypoxia and dysfunction in both male and female mice.

### 4.1 *Tert* loss causes EC senescence and dysfunction irrespective of telomere length

While it is clear that senescent EC accumulate with age and become dysfunctional, it has not been determined whether senescence is a cause or a consequence in this process. We have reported that adipocyte progenitor proliferation is promoted by increased caloric intake, which accelerates telomere attrition (Eckel‐Mahan et al., 2020; Gao et al., 2020; Ribas‐Latre et al., 2021). In EC‐*Tert*‐KO mice, HFD feeding also accelerates telomere attrition, contributing to the induction of replicative senescence. However, EC from the brain and skeletal muscle of EC‐*Tert*‐KO mice have normal telomere length, yet, still display features of senescence even in young mice. Moreover, adipose EC from mice raised on regular chow displayed the same senescence phenotype in culture as that observed for EC from mice fed HFD, consistent with comparable activation of genes coding for cell cycle inhibitors p16, p21, and p19, the effectors of senescence. Induction of EC senescence in the absence of TERT, commencing irrespective of telomere length, indicates that a non‐canonical function of telomerase is responsible for the maintenance of normal EC physiology and proliferative capacity.

Much of what is known about TERT function is based on studies in cancer cells, in which it is often re‐activated and promotes tumor growth by enabling enzymatic activity of telomerase (Blackburn et al., 2006). A number of studies demonstrated that TERT has telomere‐independent effects on cell transcriptome and physiology (Romaniuk et al., 2019). In addition to its catalytic function in the shelterin complex, *Tert* has been shown to regulate gene expression in cancer cells (Chakravarti, Lee, et al., 2021). Its global effect on the transcriptome is apparent from ectopic expression experiments (Mojiri et al., 2021). The inducing effect of TERT on the Wnt/β‐catenin pathway, which activates cell division and differentiation (Dratwa et al., 2020), is likely to partly explain the proliferative defect of *Tert*‐null EC and the aberrant conversion of EC into adipocytes that we observed. In cancer cells, *Tert* has also been reported to activate the NF‐κB pathway (Ghosh et al., 2012). Instead, in *Tert*‐null EC, we observed upregulation of the NF‐κB pathway. Because NF‐κB inflammatory signaling is pivotal in the SASP, it may be an indirect result of the cell senescence pathway induction. *Tert* contains a mitochondria‐localization signal, and it has been shown that mitochondrial *Tert* trafficking is important for its cytoprotective effects in EC (Ait‐Aissa et al., 2022; Ale‐Agha et al., 2021). Molecular mechanisms of *Tert* function in mitochondria remain to be elucidated. Oxidative stress marker upregulation in *Tert*‐KO EC, detected by RNAseq in our study, indicates the elevation of reactive oxygen species (ROS). Consistent with our results, inactivation of telomeric repeat‐binding factor 2 (TRF2) in EC also resulted in increased ROS (Barinda et al., 2020; Bloom et al., 2023). These findings reiterate the notion that Tert contributes to the protection of mtDNA from oxidative damage by mitigating mitochondrial ROS levels (Dratwa et al., 2020). Mitochondrial dysfunction is sufficient to induce cell senescence (Wiley et al., 2016). Therefore, mitochondrial insufficiency, occurring in the absence of *Tert*, may account for many components of the compound phenotype observed in EC‐*Tert*‐KO mice.

### 4.2 Molecular mechanisms mediating EC dysfunction caused by *Tert* inactivation

RNAseq data provide insights into the mechanisms linking ROS elevation and cell senescence induction with EC dysfunction and hypoxia response observed in the absence of *Tert*. Hypoxia‐induced vascular remodeling is known to be mediated by EC senescence (Kyi et al., 2022). The pathways most highly activated in *Tert*‐KO EC center around HIF1a, which includes its epigenetic activator HDAC4 (Geng et al., 2011), as shown in Figure S2c. It has been reported that chronic hypoxia is linked with changes in mitochondrial composition and function (Dong & Tsai, 2023). HIF1a has been established to play a specific role in this adaptation (Chaillou, 2018). Hypoxia is known to increase ROS levels (Tafani et al., 2016). Indicating a positive feedback loop, increased ROS activate *Hif1a* transcription though NF‐κB (Bonello et al., 2007). *Tp53*, the master cell cycle checkpoint gene, which we consistently found upregulated in *Tert* KO EC, is known to be induced by hypoxia and, specifically, by HIF1a (Sermeus & Michiels, 2011). HIF1a has been shown to engage the glycolytic cascade (Basse et al., 2017), which explains the metabolic phenotype observed. Because *Tp53* induction also leads to HIF1a stabilization, *Tert* inactivation may activate a positive regulatory loop boosting HIF1a activity. Markers of vasoconstriction upregulated in *Tert* KO EC (Figure S2b,c) are also indicative of increased HIF1a signaling (Lim et al., 2013) and consistent with reduced blood vessel dilation linked with EC senescence in other studies (Bloom et al., 2023; Jia et al., 2019). Other genes found as hallmarks of EC dysfunction in *Tert* KO EC by the IPA analysis provide additional clues on the function of TERT (Figure S2a–c). Upregulation of angiotensinogen (*Agt*), observed for *Tert*‐KO EC, is also predictive of vasoconstriction. The effects of AGT are executed by angiotensin II and reported to cause senescence of vascular smooth muscle cells (Kunieda et al., 2006). Another upregulated AGT messenger is anoctamin 1 (ANO1), a calcium‐activated chloride channel implicated in ROS production in EC due to mitochondrial membrane potential mismanagement. Downstream on ANO1, activation of *Notch1* signaling (Figure S2b) induces *Ptgs2* expression, detected in *Tert* KO EC. *Ptgs2*, coding for COX2, has been reported to regulate the expression of SASP components (Goncalves et al., 2021). Consistent with this, NOTCH1 has also been identified as a switch of secretome patterns during senescence induction (Hoare et al., 2016). Downstream of *Notch1*, upregulation of *Akt1* detected in *Tert*‐KO EC, is likely to be key in senescence induction. The induction of p53, p16, and p19 requires AKT1 activity (Nogueira et al., 2008). In addition, expression of an anti‐apoptotic factor BCL‐2, key in senescence, is directly activated by AKT1 signaling (Pugazhenthi et al., 2000). Another induced gene, *Hmgcr* (HMG‐CoA reductase), also downstream of *Akt1*, is another effector of senescence (Assmus et al., 2003). How these pathways are initially activated by *Tert* loss is not completely clear. However, the hypoxia response is likely to be a trigger. As expected, in EC of KO mice had a 9.6‐fold higher (*p* = 1.87 × 10^−12^), expression of *Vegfa*, an angiogenic factor upregulated by hypoxia.

### 4.3 Bystander effects of endothelial *Tert* inactivation on other cells

As revealed by scRNAseq in our study, EC *Tert* KO induces gene expression changes in other cells of AT, which are indicative of secondary senescence. Genomics data are consistent with ASC from KO mice displaying abnormal morphology in cell culture (Figures 1h and 4d). Induction of NOTCH1 signaling, known to mediate intercellular interaction, in *Tert*‐KO EC may be one of the mechanisms transducing the bystander effect. Importantly, transcriptional changes in ASCs were overall similar to those observed in EC. As in EC, the primary changes detected were linked with mitochondrial dysfunction and hypoxia response. Specifically, the CLEAR (coordinated lysosomal expression and regulation) network, the most highly upregulated (Figure S3a), involves autophagy, which is promoted by HIF1a (Palmieri et al., 2011). Pro‐fibrotic *TGFb1‐Smad3* signaling, upregulated in *Tert* KO EC (Figure S2c), as well as in ASCs (Figure S3a) is also positively linked with HIF1a activity. A similar bystander effect of EC senescence was observed in ASCs, SMCs, and leukocytes. Some of these effects could be induced by deep tissue hypoxia impinging on all organ‐composing cells. However, we also observed that *Tert*‐null EC induced SA‐β‐gal in differentiated 3 T3‐L1 adipocytes in cell culture (data not shown). A similar observation was made for another EC senescence model, based on a dominant negative (DN) form of TRF2 (Barinda et al., 2020). This suggests that the induction of senescence markers in adjacent *Tert* + cells is caused by the SASP factors (Barinda et al., 2020). Interleukin IL1a has been identified as a key trigger of EC senescence for the DN TERF2 model (Barinda et al., 2020). This particular SASP factor was not upregulated in EC by *Tert* deletion in our study, suggesting that the senescence phenotype can accompany the dysfunction of EC achieved via distinct mechanisms. Results from various endothelium‐specific manipulations suggest that the cell senescence phenotype may be a manifestation of EC dysfunction, rather than, or as well as, a cause of their dysfunction. Changes in the immune system and the leukocyte‐EC interactions, in particular in AT, may be important in considering systemic physiological effects.

### 4.4 Effects of EC *Tert* deletion on distinct organs and systemic metabolism

Inexorable deterioration of mitochondrial function with aging leads to gradual glycolysis increase at the expense of oxidative phosphorylation, which causes progressive energy deficit contributing to impaired function of vital organs. This applies to the physiology of the CNS and muscle tissues (Serio et al., 2023). Chronic hypoxia leads to blood vessel remodeling resulting in their increased permeability (Stenmark et al., 2006). Leakiness of the vasculature also progresses with older age (Oakley & Tharakan, 2014). Another hallmark of aging is the decrease of AT mass, which normally precedes lean muscle mass loss (Caso et al., 2013). The mouse model of endothelial *Tert* KO appears to represent the attributes of aging‐related changes in physiology. Indeed, increased blood vessel permeability was observed in EC‐*Tert*‐KO mice. Our data indicate that *Tert*‐KO EC have reduced mitochondrial content and function, which results in increased dependence on glycolysis. Consistent with this, transcriptomic analysis of EC‐*Tert*‐KO mice indicated the suppression of oxidative phosphorylation.

After being fed with HFD, EC‐*Tert*‐KO mice become deficient in AT accumulation due to replicative senescence of EC. We show that in these mice the vasculature becomes replaced by cells of another lineage, which likely further jeopardizes its function. An alternative explanation of the cre‐lox system being incompletely efficient does not explain the differences between the WT and KO mice. We observed that, without *Tert*, EC are predisposed to differentiate into adipocytes, which could have different capacity to store lipids than adipocytes of the canonical mesenchymal lineages. AT in EC‐*Tert*‐KO mice was less responsive to lipolytic signals. This provides further evidence that endothelial TERT is important for multiple functions of adipose cells. Indirect calorimetry data and increased fatty acid circulation indicate that EC‐*Tert*‐KO have a reduced utilization of lipids as an energy substrate. We show that EC‐*Tert*‐KO mice have lower circulating glucose levels, due to its increased utilization for energy, as reflected by the RER. In the context of hypoxia, induced EC‐*Tert*‐KO mice, glucose utilization through glycolysis is expected, which is exactly what our data indicate. Lower circulating insulin levels in EC‐*Tert*‐KO mice may explain why they are more sensitive to insulin administration. Consistent with other mouse models of EC senescence (Barinda et al., 2020; Bloom et al., 2023) EC‐*Tert*‐KO mice have moderate glucose intolerance, a pre‐diabetic condition accompanying aging (Chia et al., 2018). The metabolic phenotype was not prominent in *Tert*‐EC‐KO mice raised on regular chow. Notably, EC senescence models based on TRF2 inactivation displayed a slightly different phenotype: elevated blood insulin and glucose intolerance, which developed in older mice even on regular diet (Barinda et al., 2020). Insulin intolerance observed upon EC TRF2 inactivation (Bloom et al., 2023) was also not observed for EC‐*Tert*‐KO mice. The nuanced phenotypic distinctions may due to *Tert*‐EC‐KO mice displaying reduced adiposity, unlike the EC TRF2 inactivation models. Alternatively, unique functions of TERT and TRF2 may affect cell physiology differently in addition to preventing senescence.

Another hallmark of aging is skeletal muscle dysfunction (Wilkinson et al., 2018). Skeletal muscle contributes to the changes in the glucose/lipid energy balance in EC‐*Tert*‐KO mice. Senescence pathways were activated in *Tert*‐KO EC isolated from the muscle despite the lack of a proliferative defect. While sarcopenia was not observed, EC‐*Tert*‐KO mice had reduced exercise endurance. Thus, increased fatigue resistance observed in the elderly (Siparsky et al., 2014) is represented by this model. EC‐*Tert*‐KO mice maintained short‐term strength, likely due to increased glycolytic capacity. Nevertheless, EC‐*Tert*‐KO mice are useful for modeling age‐related changes is muscle endurance. Interestingly, a number of myogenesis genes were found to be upregulated in skeletal muscle‐derived EC of *Tert*‐EC‐KO mice. This included *Tbxt*, which is upstream of *Myf5* and *Myod*, *Rac1*, which is upstream of *Myog*, as well as *Foxo1*, *Smarcd3*, *Srf*, and *Socs1*. Upregulation of these myogenic genes, could be due to decreased expression of *Dnmt3b*, reported to be regulated by *Tert* (Yu et al., 2018). Indeed, decreased expression of *Dnmt3b* was observed in *Tert*‐KO EC in our transcriptomic analysis. A number of contractile genes were found to be downregulated in *Tert* KO muscle EC, including *Acta*, *Actn3*, *Myh1*, *Myh4*, *Myoz1*, and *Myom1*. This is likely to contribute to the decreased ability of senescent EC to vasodilate reported recently (Bloom et al., 2023), which is expected to contribute to organ dysfunction. As microenvironment aging affects myocyte function (Lu et al., 2022), the effect of EC senescence on the muscle is likely mediated by secondary dysfunction of other cells.

A progressively concerning consequence of aging is the impairment of CNS function. This relates to both, metabolism deregulation and memory loss. The prevalence of vascular dementia closely trails that of AD (Romay et al., 2019). There is accumulating evidence that cognitive decline occurs in part due to accumulation of senescent cells in the brain (Ogrodnik et al., 2019). Here, EC‐*Tert*‐KO mice were found to have a decreased brain EC proliferation and memory impairment comparable to that induced by LPS injection. It is not clear how exactly EC *Tert* inactivation and senescence interferes with memory formation and establishing this is beyond the scope of this study. However, it is tempting to speculate that the CNS dysfunction in EC‐*Tert*‐KO mice is linked with increased vascular leakiness resulting from hypoxia. It is likely that jeopardized BBB function, implicated in vascular dementia (Sweeney et al., 2018), is the cause of the phenotype observed. In addition, EC‐*Tert*‐KO mice run a chronic low fever, which partly accounts for their higher energy expenditure. Thermoregulation is known to be regulated by pyrogenic molecules crossing the BBB in the hypothalamus (Balli et al., 2023). In our study, vascular leakiness was observed in preoptic area controlling thermoregulation. Moreover, COX2 expression, regulating the production of prostaglandins causing fever (Tan & Knight, 2018), is increased in EC‐*Tert*‐KO mice. Interestingly, increased body temperature was not detected for EC‐*Tert*‐KO mice fed chow (data not shown). The dependence of fever observed on HFD feeding could be explained by arachidonic acid being the predecessor of prostaglandins. Increased access of prostaglandins and pyrogenic SASP factors to the hypothalamus due to increased BBB permeability likely accounts for the chronic low‐grade fever in EC‐*Tert*‐KO mice.

## 5 CONCLUSIONS

Our study provides evidence that *Tert* in the endothelium plays an important role in suppressing senescence and maintaining normal function of EC and the surrounding cells. It remains to be determined how closely cell dysfunction induced by TERT KO in mice simulates cell senescence arising in natural aging. However, evidence is accumulating that re‐activation of telomerase can have beneficial anti‐aging effects (Nazari‐Shafti & Cooke, 2015). In mice, *Tert* gene therapy delays aging and increases longevity without increasing cancer (Bernardes de Jesus et al., 2012). Recently, intranasally delivered *Tert* gene therapy was shown to extend mouse lifespan (Jaijyan et al., 2022). Telomere shortening and senescence have been observed in atherosclerotic areas of human vasculature, and inhibition of human telomerase results in endothelial dysfunction, reversible by TERT expression (Minamino et al., 2002). Yet, there are no conclusive human data on telomere attrition in the endothelium to link it to endothelial senescence observed in aging. Also, there is no evidence of TERT expression in somatic cells of humans, and cell dependence on it may be different in mice and humans. However, induction of TERT decreased DNA damage activation and inflammatory signaling in human colon organoids from patients with inflammatory bowel disease (Chakravarti, Lee, et al., 2021). Moreover, TERT gene therapy enhances learning pathway networks in human neurons (Shim et al., 2021). In the future, improved and safe approaches to re‐activate TERT and/or prevent its inactivation may become effective in suppressing and delaying the onset of cell senescence and aging‐associated pathologies. According to our data revealing the metabolic function of TERT, and the reports on organelle‐targeted TERT having higher efficacy in cell revitalization, approaches to reactivating telomerase specifically in mitochondria may be particularly beneficial. We conclude that EC‐*Tert*‐KO present a “humanized” mouse model of EC aging that can be used for studying mechanisms of aging‐associated organ dysfunction. This model can also be useful to study the effects of treatments activating TERT expression and could help further dissect the benefits of canonical and non‐canonical TERT function.

## AUTHOR CONTRIBUTIONS

Conceptualization: KEM and MGK. Animal and cell experimentation: ZG, RBS, and YY. Data analysis: ZG, RBS, YY, JR, RVD, KEM, and MGK. Writing—original draft: MGK. Writing, review, and editing: All other authors. Funding acquisition: KEM and MGK. All authors contributed to the article and approved the submitted version.

## FUNDING INFORMATION

This research was supported by the NIH grant 1R01DK125922, CPRIT grant RP180734, the Levy‐Longenbaugh Fund, and the Bovay Foundation.

## CONFLICT OF INTEREST STATEMENT

The authors declare no conflict of interest.

## Supporting information


Figure S1.
Figure S2.
Figure S3.

Data S1:

## Data Availability

The data that support the findings of this study are available from the corresponding author upon request. The datasets generated for this study can be found in the GEO database. Total RNA‐seq: GSE239686 (Token: shifuooipzapzwr); single‐cell RNA‐seq: GSE239687 (Token: gxqdeiugjjibnin).
