# Supplementary material for: Endothelial‐specific telomerase inactivation causes telomere‐independent cell senescence and multi‐organ dysfunction characteristic of aging
Source: Aging Cell. 2024 Mar 12;23(6):e14138. doi: 10.1111/acel.14138 (PMC11296101; doi:10.1111/acel.14138)
Supplement: Supplementary file 1 — Figure S1. Figure S2. Figure S3. [file ACEL-23-e14138-s002.pdf]

a

Most downregulated

gene_name	gene_biotype	gene_description	log2 FoldChange	p value	Gene function suggesting a role in Tert KO response
Inpp5j	protein_coding	inositol polyphosphate 5-phosphatase J [Source:MGI Symbol;Acc:MGI:2158663]	-9.858488889	3.16E-15	Promotion of angiogenesis
Il17rc	protein_coding	interleukin 17 receptor C [Source:MGI Symbol;Acc:MGI:2159336]	-9.858488889	3.16E-15	Promotion of angiogenesis
Nrn1	protein_coding	neuritin 1 [Source:MGI Symbol;Acc:MGI:1915654]	-9.858488889	3.16E-15	EC migraiton
Rom1	protein_coding	rod outer segment membrane protein 1 [Source:MGI Symbol;Acc:MGI:97998]	-9.911544053	1.57E-15	Adhesion
Celf4	protein_coding	CUGBP, Elav-like family member 4 [Source:MGI Symbol;Acc:MGI:1932407]	-9.911544053	1.57E-15	RNA-binding, alternative splicin
Myo1d	protein_coding	myosin ID [Source:MGI Symbol;Acc:MGI:107728]	-9.928804211	1.12E-15	Cell-cell signaling and cell migration
1700019D03Rik	protein_coding	RIKEN cDNA 1700019D03 gene [Source:MGI Symbol;Acc:MGI:1914330]	-9.945860311	9.45E-16	Predicted to regulate PKA
Vegfc	protein_coding	vascular endothelial growth factor C [Source:MGI Symbol;Acc:MGI:109124]	-9.96271712	7.99E-16	Lymph-angiogenesis
Pnma1	protein_coding	paraneoplastic antigen MA1 [Source:MGI Symbol;Acc:MGI:2180564]	-9.96271712	7.99E-16	Cell viability, inhibits apoptosis
Ms4a7	protein_coding	membrane-spanning 4-domains, subfamily A, member 7 [Source:MGI Symbol;Acc:MGI:1918846]	-9.979379243	5.74E-16	Signal transduction
Ipp	protein_coding	IAP promoted placental gene [Source:MGI Symbol;Acc:MGI:96581]	-9.995851126	4.87E-16	Actin-binding
Tmem237	protein_coding	transmembrane protein 237 [Source:MGI Symbol;Acc:MGI:2138365]	-10.01213706	3.51E-16	Cilia
Dusp14	protein_coding	dual specificity phosphatase 14 [Source:MGI Symbol;Acc:MGI:1927168]	-10.05992003	1.86E-16	VEGF-regulated
Pard6g	protein_coding	par-6 family cell polarity regulator gamma [Source:MGI Symbol;Acc:MGI:2135606]	-10.09091817	1.36E-16	Cell polarity, tight junctions
Lrrc18	protein_coding	leucine rich repeat containing 18 [Source:MGI Symbol;Acc:MGI:1914830]	-10.106171	9.96E-17	Marker of coronary artery disease
Flrt2	protein_coding	fibronectin leucine rich transmembrane protein 2 [Source:MGI Symbol;Acc:MGI:3603594]	-10.12126426	8.55E-17	Inter-endothelial adhesion
Klf8	protein_coding	Kruppel-like factor 8 [Source:MGI Symbol;Acc:MGI:2442430]	-10.12126426	8.55E-17	VEGFA and Cyclin D activator
0610039K10Rik	antisense	RIKEN cDNA 0610039K10 gene [Source:MGI Symbol;Acc:MGI:1915636]	-10.12126426	8.55E-17	Unknown
A630072L19Rik	antisense	RIKEN cDNA A630072L19 gene [Source:MGI Symbol;Acc:MGI:3641830]	-10.12126426	8.55E-17	Unknown
**Tert**	protein_coding	telomerase reverse transcriptase [Source:MGI Symbol;Acc:MGI:1202709]	-10.36898724	2.45E-18	**Telomerase reverse transcriptase**
Mcf2l	protein_coding	mcf.2 transforming sequence-like [Source:MGI Symbol;Acc:MGI:103263]	-10.41870329	1.27E-18	G exchange Rho/Rac co-factor
Slc24a1	protein_coding	solute carrier family 24, member 1 [Source:MGI Symbol;Acc:MGI:2384871]	-10.43086915	9.85E-19	Ca/K transport
Actn3	protein_coding	actinin alpha 3 [Source:MGI Symbol;Acc:MGI:99678]	-10.65484207	3.89E-20	Muscle metabolism
Igkv6-15	IG_V_gene	immunoglobulin kappa variable 6-15 [Source:MGI Symbol;Acc:MGI:1330831]	-10.79320696	5.03E-21	Immunoglobulin kappa variable chain

Most upregulated

gene_name	gene_biotype	gene_description	log 2FoldChange	p value	Gene function suggesting a role in Tert KO response
Selenoo	protein_coding	selenoprotein O [Source:MGI Symbol;Acc:MGI:1919007]	11.02069037	1.75E-20	Redox activity, mitochondrial function
Mir6395	miRNA	microRNA 6395 [Source:MGI Symbol;Acc:MGI:5530943]	10.50034444	3.00E-17	Reduced by LPS treatment
Itga3	protein_coding	integrin alpha 3 [Source:MGI Symbol;Acc:MGI:96602]	10.48197868	3.79E-17	Immune cell infiltration
Ccdc34	protein_coding	coiled-coil domain containing 34 [Source:MGI Symbol;Acc:MGI:1915451]	10.16644961	2.85E-15	Proliferation
Zfyve21	protein_coding	zinc finger, FYVE domain containing 21 [Source:MGI Symbol;Acc:MGI:1915770]	10.16644961	2.85E-15	Transcription factor
Zcchc10	protein_coding	zinc finger, CCHC domain containing 10 [Source:MGI Symbol;Acc:MGI:1196228]	10.11969678	5.00E-15	Transcription factor
Snhg6	lincRNA	small nucleolar RNA host gene 6 [Source:MGI Symbol;Acc:MGI:1921074]	10.1117552	5.76E-15	Modulates oxidized LDL-induced endothelial cell injury
Paqr8	protein_coding	progestin and adipoQ receptor family member VIII [Source:MGI Symbol;Acc:MGI:1921479]	10.08766471	7.67E-15	arrests endothelial cell cycle
Zbtb8a	protein_coding	zinc finger and BTB domain containing 8a [Source:MGI Symbol;Acc:MGI:1920930]	10.04659783	1.38E-14	Transcription factor
Ptp4a1	protein_coding	protein tyrosine phosphatase 4a1 [Source:NCBI gene;Acc:19243]	10.02983807	1.60E-14	Suppressor of vascular inflammation
Ldhd	protein_coding	lactate dehydrogenase D [Source:MGI Symbol;Acc:MGI:106428]	10.00432762	2.50E-14	Glycolysis
Rdh5	protein_coding	retinol dehydrogenase 5 [Source:MGI Symbol;Acc:MGI:1201412]	9.960781441	3.95E-14	Lipid metabolism
Adcy5	protein_coding	adenylate cyclase 5 [Source:MGI Symbol;Acc:MGI:99673]	9.831357826	2.28E-13	Generation of cAMP, linked with decreased longevity
Aspm	protein_coding	abnormal spindle microtubule assembly [Source:MGI Symbol;Acc:MGI:1334448]	9.79214208	3.18E-13	Cell division
Ndnf	protein_coding	neuron-derived neurotrophic factor [Source:MGI Symbol;Acc:MGI:1915419]	9.782169292	3.77E-13	Ischemia-induced revascularization
C7	protein_coding	complement component 7 [Source:MGI Symbol;Acc:MGI:88235]	9.772127085	4.46E-13	Trap for assembling terminal complement complex
Pla2g12a	protein_coding	phospholipase A2, group XIIA [Source:MGI Symbol;Acc:MGI:1913600]	9.762014487	5.29E-13	Fatty acid synthesis, injures lymphatic endothelium
Alg11	protein_coding	asparagine-linked glycosylation 11 (a-1,2-mannosyltransferase) [Source:MGI Symbol;Acc:MGI:2142632]	9.762014487	5.29E-13	Glycosylation
Hist2h2ac	protein_coding	histone cluster 2, H2ac [Source:MGI Symbol;Acc:MGI:2448316]	9.762014487	5.29E-13	Nucleosome
Zfp975	protein_coding	zinc finger protein 975 [Source:MGI Symbol;Acc:MGI:3648690]	9.751830504	5.29E-13	Transcription factor
Clspn	protein_coding	claspin [Source:MGI Symbol;Acc:MGI:2445153]	9.731244301	8.03E-14	Genotoxic stress signaling, DNA replication
Fam171b	protein_coding	family with sequence similarity 171, member B [Source:MGI Symbol;Acc:MGI:2444579]	9.731244301	8.03E-14	Stabilizes vimentin and enhances TAM infiltration
Rragb	protein_coding	Ras-related GTP binding B [Source:MGI Symbol;Acc:MGI:3038613]	9.720839985	9.58E-14	Guanine-binding protein
Ntpcr	protein_coding	nucleoside-triphosphatase, cancer-related [Source:MGI Symbol;Acc:MGI:1913816]	9.71036009	9.58E-14	Anti-oxidant enzyme activated by oxidative stress

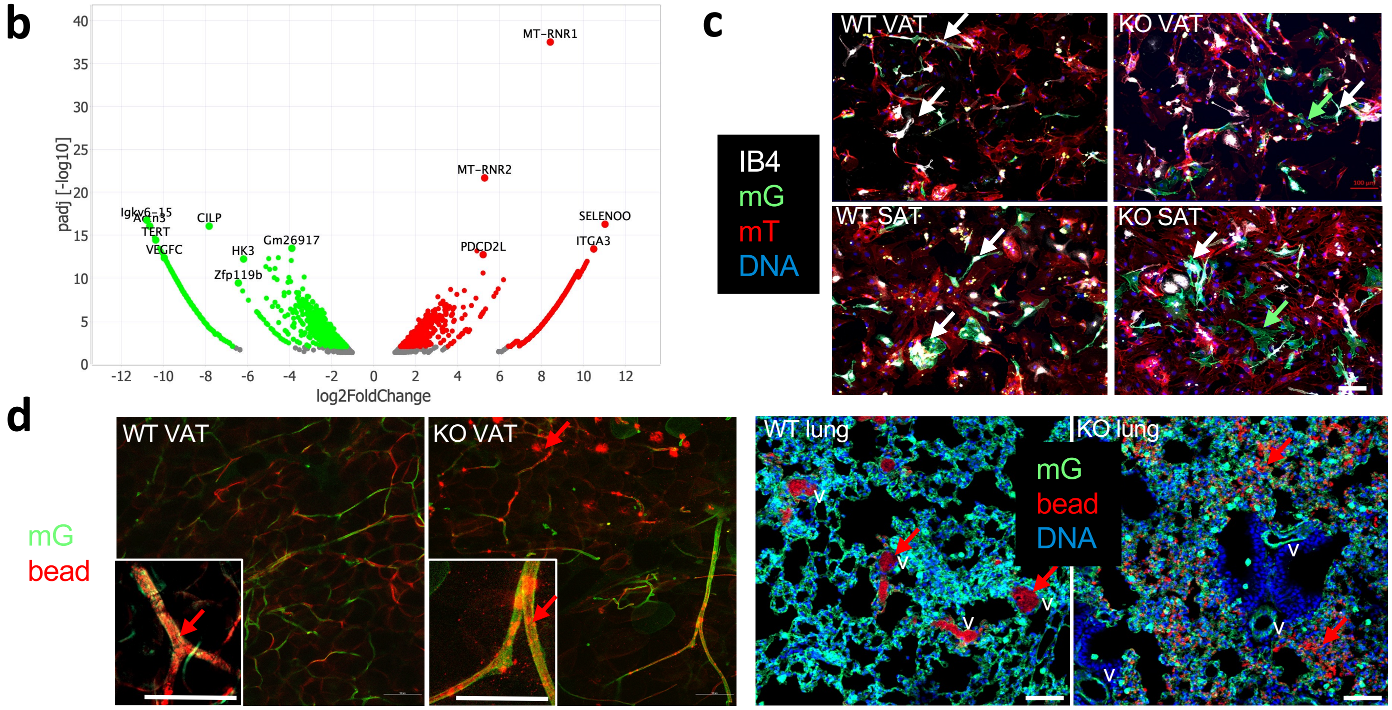

Supplementary Figure S1. (a-b) RNAseq analysis on mG+ isolated from SAT of KO vs WT littermates raised on HFD. Data are reported in Gao et al., *Front Cell Dev Biol* 2023 and deposited in GEO (accession GSE239686, Token: shifuooipzapzwr). Listed are the most KO-downregulated and KO-upregulated 24 genes. (b) Volcano plot (IPA) demonstrates *Tert* to be among the most downregulated genes in SAT EC of KO mice, validating knockout efficiency. (c) SVC from WT and KO VAT and SAT stained with isolectin B4 (IB4) showing that some of the KO mG+ cells are IB4-negative and have fibroblast, rather than EC, morphology. (d) After injection of 200 μl of 0.2% red fluorescent FluoSphere (Invitrogen F8801) microbeads (bead), 30 min later VAT whole mounts (left) and lung frozen sections (right) were analyzed for bead distribution. Arrows: bead fluorescence inside WT vessels (v) and outside (KO). Scale bar: 100 μm.

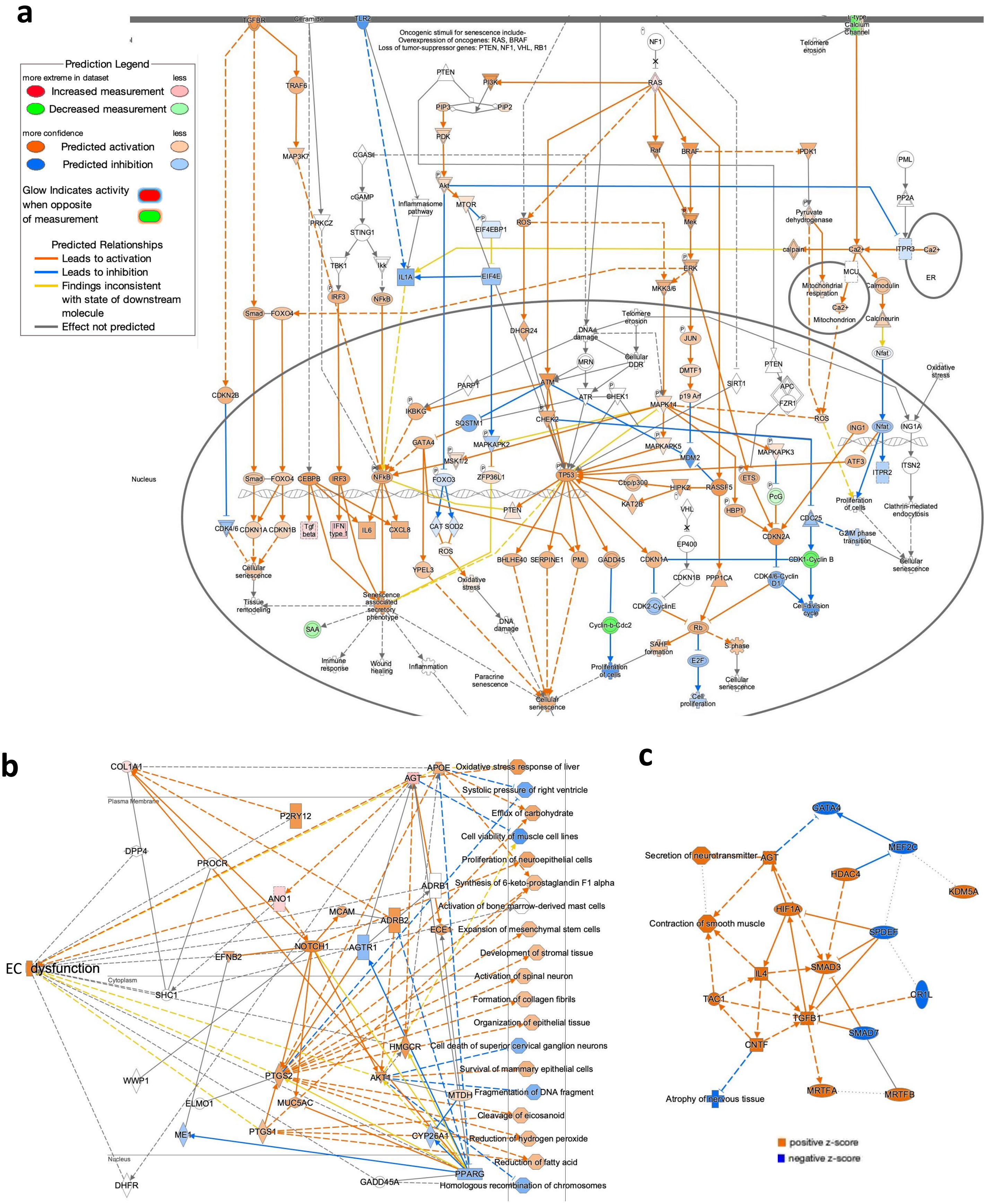

Supplementary Figure S2. EC *Tert* KO results in adipose EC senescence and dysfunction, as detected by Ingenuity Pathway Analysis (IPA) of RNAseq data available at GEO database (accession GSE239686, Token: shifuooipzapzwr) and reported in Gao et al., *Front Cell Dev Biol* 2023. (a) Senescence-related pathways upregulated in VAT EC of EC-TERT KO mice fed chow, compared to WT EC. (b) EC dysfunction-related pathways upregulated in VAT EC of EC-TERT KO mice fed chow, compared to WT EC. (c) Top canonical pathways deregulated in VAT EC of EC-TERT KO mice fed chow, compared to WT EC.

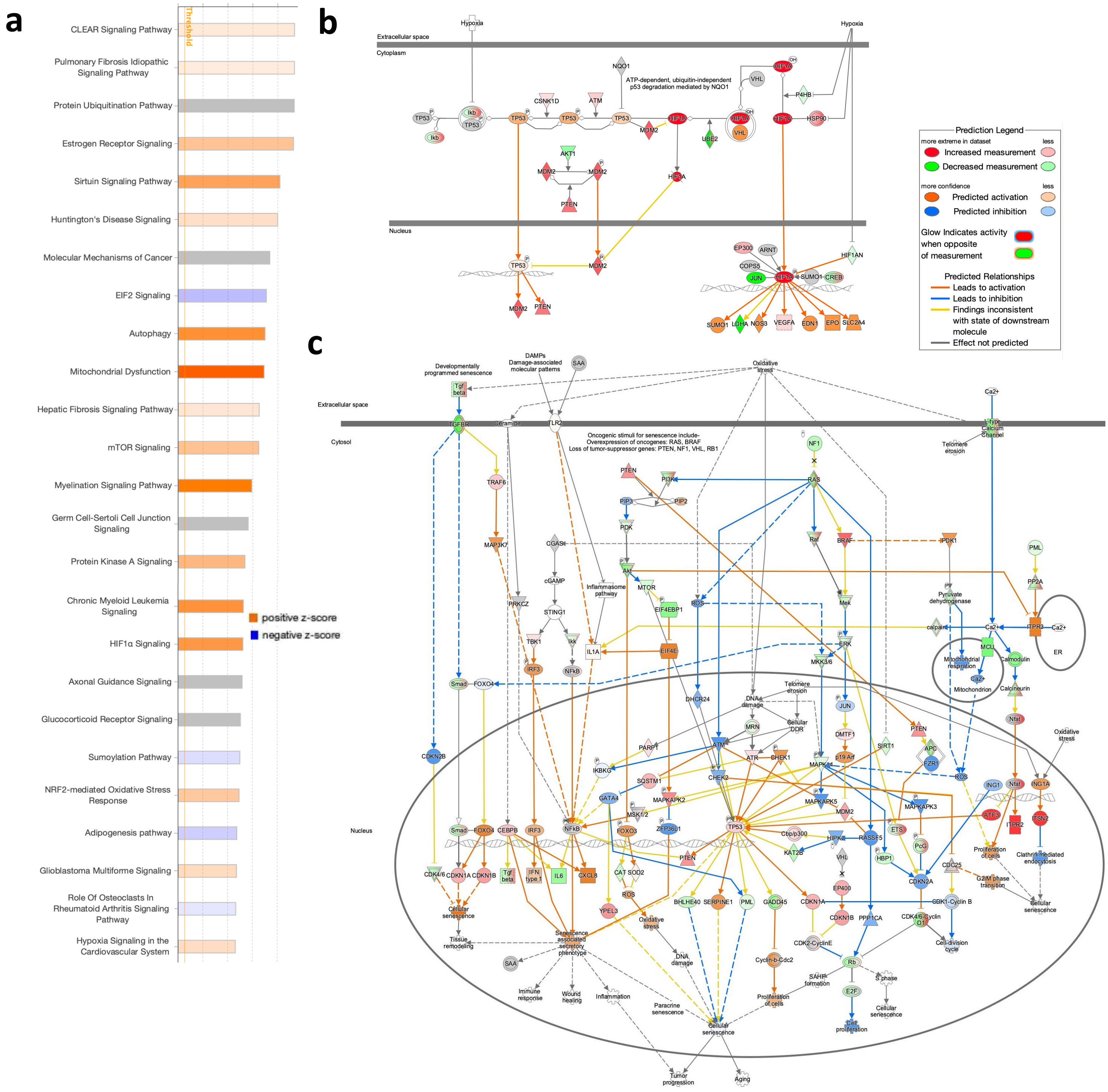

Supplementary Figure S3. EC *Tert* KO-induced hypoxia affects other cells in AT, as determined by scRNAseq on SAT of EC-*Tert*-KO and WT mice fed HFD for 5 months as described and reported in Gao et al., *Front Cell Dev Biol* 2023. cDNAs were generated using oligo-T primers without rRNA depletion. Single cell capture and library construction were performed with the Chromium Single Cell 3' Reagent Kit v3.1. Sequencing was done with Illumina NextSeq 550 System using High Output Kit v2.5 (50,000 reads / cell). Cell Ranger Single Cell Software Suite v.3.1.0 was used to perform bioinformatic analysis against mouse transcriptome (mm10, Ensembl 93) with STAR. Raw read count tables were analyzed using the Seurat (v3.1.1) on R platform (3.5.2). FindVariableGenes was used to calculate the principal components. Cell clusters were identified using the Shared Nearest Neighbor (SNN) algorithm with a resolution parameter 0.8. Transcriptomes of adipose stromal cells (ASC), were compared by IPA. (a) A list of signaling networks activates (orange) or inactivated (blue) in ASC from SAT of EC-*Tert*-KO mice. (b) Analysis focusing on hypoxia-related pathways showing upregulation of senescence markers in ASC from VAT of EC-*Tert*-KO mice. (c) Analysis focusing on senescence-related pathways showing upregulation of senescence markers in ASC from SAT of EC-*Tert*-KO mice. Full data are available at GEO database (accession GSE239687, Token: gxqdeiugjjibnin).
